# Retrospective Longitudinal Monitoring of Multiple Myeloma Patients by Mass Spectrometry Using Archived Serum Protein Electrophoresis Gels and De Novo Sequence Analysis

**DOI:** 10.1097/HS9.0000000000000758

**Published:** 2022-08-02

**Authors:** Somayya Noori, Marina Zajec, Henk Russcher, Andrei N. Tintu, Annemiek Broijl, Joannes F. M. Jacobs, Theo M. Luider, Yolanda B. de Rijke, Martijn M. vanDuijn

**Affiliations:** 1 Department of Neurology, Erasmus MC, University Medical Center, Rotterdam, The Netherlands; 2 Department of Clinical Chemistry, Erasmus MC, University Medical Center, Rotterdam, The Netherlands; 3 Department of Hematology, Erasmus MC, University Medical Center, Rotterdam, The Netherlands; 4 Department of Laboratory Medicine, Radboud University Medical Center, Nijmegen, The Netherlands

Detection of M-protein, a monoclonal immunoglobulin produced by malignant plasma cells in the bone marrow, is crucial in multiple myeloma (MM) diagnostics, treatment, and follow-up.^1^ M-protein is most commonly detected and quantified with serum protein electrophoresis (SPEP) using patient serum or urine as the starting material for analysis. In a healthy individual, the gamma fraction of the SPEP gel is broad and diffuse, as it contains a polyclonal immunoglobulin repertoire.^2^ In MM, a sharp band called an M-spike appears due to high abundance and monoclonality of the M-protein relative to the polyclonal background.^3^

SPEP has a relatively limited sensitivity and cannot detect M-protein lower than 0.5 g/L.^4^ With new treatment strategies, an increasing percentage of MM patients obtain stringent complete remission.^5^ SPEP and immunofixation electrophoresis (IFE) are not sensitive enough to monitor such deep responses in MM. More sensitive techniques exist to monitor minimal residual disease (MRD) in MM, such as flow cytometry and next generation sequencing, but all of these require invasive bone marrow sampling.^6^ With mass spectrometry (MS), it is possible to monitor M-proteins in blood in a more sensitive manner than with SPEP.^7-9^

Our group has previously developed a sensitive blood-based MS assay for monitoring of M-protein-specific clonotypic peptides in M-protein bands isolated from routine SPEP gels.^10^ This M-protein detection method is 100 times more sensitive than routine diagnostics in blood. However, to select patient-specific M-protein peptides, RNA data from an initial bone marrow sample was used to obtain sequence information. In the present study, we have performed de novo sequencing on mass spectra to derive the M-protein sequence information directly from archived diagnostic SPEP gels without any RNAseq reference data.^11^ This study, which was approved by the institutional review board (MEC-2019-0342), demonstrates the feasibility of this approach on longitudinal samples of 9 MM patients, and demonstrates that the method allows for an earlier detection of relapses.

MM patients (n = 9, Suppl. Table S1) were retrospectively selected based on the data in the hospital information system. Patients were selected to have SPEP detectable M-protein at diagnosis (>1 g/L), and at least 1 period when the M-protein was not found, followed by again detectable M-protein. The M-proteins migrated into either the beta-region or the gamma-region of the SPEP gels. Visible or invisible M-protein bands were excised from the dehydrated gels, digested with trypsin, and the resulting peptides were extracted from the gel. M-protein specific peptides were selected from data-dependent mass spectrometry analysis of the first diagnostic sample of each patient, based on unique presence in the patients and homology of the de novo sequence to immunoglobulin germline sequences. The M-protein clonotypic peptide was subsequently quantified in all samples by targeted mass spectrometry and analysis with Skyline software.^12^ MS data as well as patient information and procedures are provided in Supplemental Digital Content (Suppl. Methods, Suppl. Tables S2-S4, Suppl. Figure S3) and in a public data repository (https://panoramaweb.org/MSMRDGelStudy.url).

At least 1 patient-specific M-protein peptide was identified for all selected MM patients using de novo sequencing and no patients were excluded due to failing this step, showing 100% feasibility of the method on the sample set (Suppl. Table S3). Figure 1 shows as an example 2 gels from patient 7 with a visible and an invisible M-protein band, an outline of the band excised, and de novo sequencing results as used for peptide selection in this patient. From the invisible SPEP gel band, the same peptides were also readily detected with MS.

**Figure 1.**
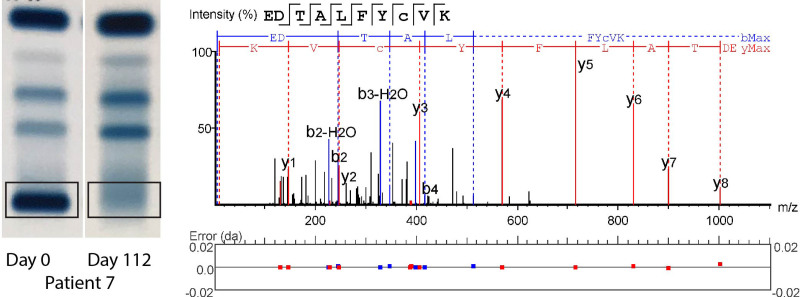
**SPEP gels and de novo sequencing.** Two representative SPEP gels are shown from patient 7, 1 positive for M-protein, the other negative. The gel band that was excised for mass spectrometry analysis has been indicated. The result of de novo sequence analysis on the first gel sample is shown, indicating fragment ion peaks associated with the amino acid sequence of the peptide and mass errors. Other peptides in Suppl. Figure S1. SPEP = serum protein electrophoresis.

The patient-specific M-protein peptides were used as surrogates for longitudinal M-protein monitoring during the disease course of each patient. Targeted MS was used to measure the M-protein-specific peptides and to compare performance with routine electrophoresis for M-protein monitoring. The results were summarized in Figure 2, which shows that the MS assay could detect the M-protein at most time points where the routine electrophoresis analysis could not. For example, patient 1 had 2 periods in the disease course when the M-protein was not detectable with electrophoresis (from days 265–485 to 938–1259), while M-protein was always detected by MS. In these 2 periods, a rise in M-protein levels can be observed by MS, 82 and 246 days before the rise was also detected by electrophoresis. With flow cytometry at 10^-4^ sensitivity, a negative MRD assessment was made in bone marrow at day 973, while M-protein remained detectable, initially decreasing followed by an increase starting at day 1073. Patient 4 is the only example in the study where the MS signal was lost during remission until the start of a relapse. In all patients, signal quality was assessed by dot product of the sample spectrum with a reference mass spectrum as illustrated in Suppl. Figure S2. In several patients, a break in M-protein trends can be observed when treatments are started, stopped, or replaced by maintenance treatment. For example, in patient 3 M-protein starts to rise again once the initial treatment is replaced with a maintenance treatment. Thus, our approach could give retrospective insights into the consequences of treatment decisions that were made for these patients and show the potential for clinicians to better track the disease over time, and also to obtain actionable information based on treatment responses.

**Figure 2.**
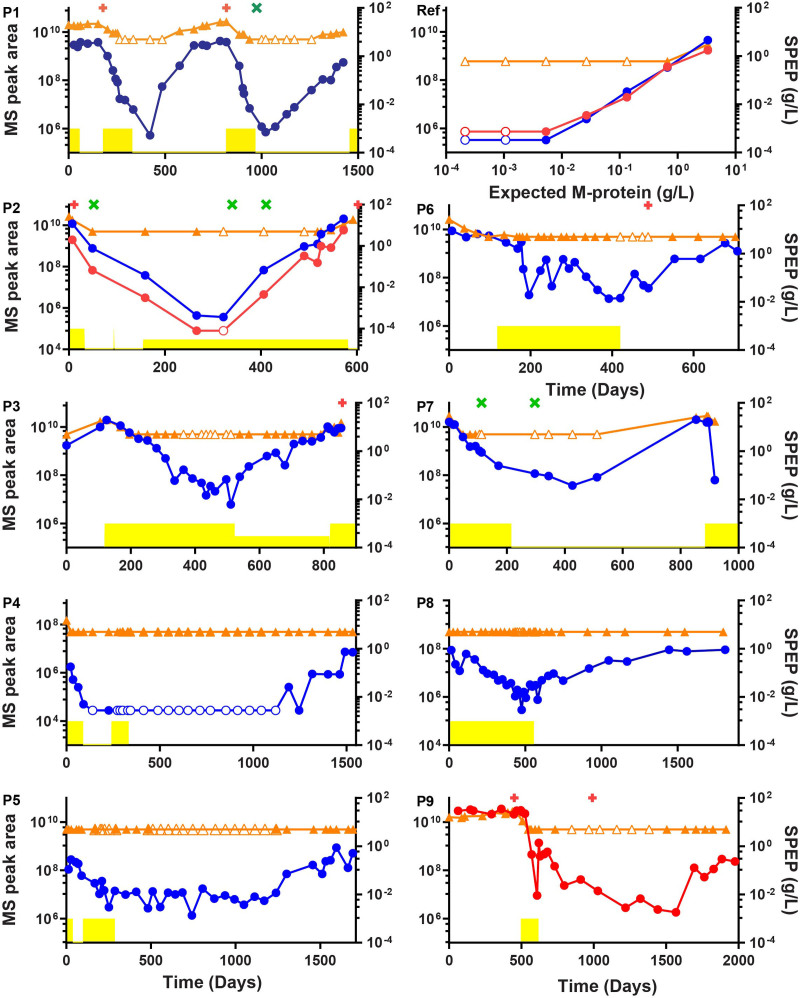
**Comparison of M-protein monitoring by routine electrophoretic techniques (serum protein electrophoresis and immunofixation electrophoresis) and the mass spectrometry assay.** Routine M-protein monitoring is shown in orange. M-protein concentration ≤5 g/L is plotted at 5 g/L, SPEP data for patient 1 includes interference from the β region. MS signals are indicated in arbitrary units and shown in blue for heavy chain peptides, and in red for the light chain. Empty symbols indicate that the M-protein/M-protein peptide was not detectable and were plotted at the level of the lowest positive sample. Patient treatment periods are shown in yellow, maintenance periods are plotted at a lower height. Bone marrow flow cytometry data are plotted as a green cross (MRD negative) or as a red plus (MRD positive). Panels P1–P9 shows the 9 patients and the panel marked Ref represents a dilution series based on the reference patient sample. MRD = minimal residual disease.

M-protein levels in blood are determined by production in the myeloma cells but also by clearance rates. The typical half-life for IgG is about 21 to 25 days, and even after an immediate removal of myeloma cells, M-protein would be cleared no faster than that rate.^13^ The decreases seen in patients 2, 3, and 7 are consistent with such typical clearance for IgG, while faster clearance may be seen for other classes of M-protein (IgA). Increases in M-protein or reduced clearance rates may relate to progressive disease or residual production. With frequent longitudinal samples, an analysis of M-protein dynamics throughout the course of disease thus becomes feasible.

De novo sequencing of a reference patient with an available M-protein heavy chain DNA sequence was performed the same way as for the selected patients. De novo sequencing was successful with 3 patient-specific M-protein peptides; 2 from the heavy chain and one from the light chain of the M-protein. Heavy chain peptide GLEWVSYLSSGGGSTYYADSVK differs from the translated DNA sequence only in amino acids leucine (L) and isoleucine (I) at position 8, as these isobaric residues are typically not distinguished in MS data. In the other de novo sequenced peptide EDTAAVYYCVR, the correct amino acids were called but not all placed at the correct position (Suppl. Table S5). However, the remaining inaccuracies in the de novo sequence do not interfere with monitoring the associated signals in the mass spectrometer, as the transitions used for targeted measurements remain the same for this identified feature and the sequence information is sufficient to establish a relation to immunoglobulins.

Serum dilutions of this reference patient were also analyzed on SPEP gels and subsequently by MS. It was found that MS can detect as low as 5 mg/L of the M-protein by monitoring patient-specific M-protein peptides. Without the use of SPEP gels, we previously reported less than 1 mg/L for the same serum samples after affinity enrichment and MS, suggesting the recovery of patient material from archived gels comes with a small loss in sensitivity due to the amount loaded on the gel and the different handling of the samples compared with cryopreserved serum samples.^14^ No visible M-protein band was detected with SPEP when the concentration of the M-protein was less than 664 mg/L.

In the current study, MS was applied to SPEP gel samples as input material. The SPEP analysis is not a necessary sample pretreatment for the MS approach as serum samples would be adequate for an equivalent analysis. Nevertheless, SPEP gels can be an attractive preanalysis for the clonotypic MS test. It enables retrospective analysis of patients in the clinic or from completed clinical trials, even when frozen serum samples are no longer available. The gels are often archived for years and tolerate non-conditioned storage. Although SPEP by itself lacks the analytical sensitivity to monitor low levels of M-protein, in combination with MS the M-protein can be monitored longitudinally with high sensitivity, and with complementary value to bone marrow methods that are invasive and can be biased by patchy disease distribution. The specific expertise required for the method might initially be provided in a centralized approach.

Future research should focus on monitoring disease progression prospectively and on absolute rather than relative quantification of the M-protein peptides. Stable isotope labeled synthetic peptides cannot be used as internal standards due to possible remaining errors in the de novo sequence. Instead, the peptide signal will have to be related to a reference sample, such as an SPEP-quantified sample from the same individual, or to a known amount of heavy-isotope labeled protein (such as SiluMab) spiked into the sample.^15^ In addition to higher sensitivity, the added value of blood-based MS analysis for MM follow-up is in more frequent MRD monitoring with minimal discomfort and may thus give new opportunities to evaluate and act on disease progression in a personalized fashion.

## AUTHOR CONTRIBUTIONS

SN and MZ did MS experiments, data analysis, and writing. HR did patient selection and SPE evaluation. ANT did patient and sample selection. AB did patient selection and clinical information. JFMJ and TML did research design and writing. YBDR did research design, patient and sample selection. MMVD did research design, data analysis, and writing.

## SOURCES OF FUNDING

JFMJ received a grant from the Dutch Cancer Society (KWF Kankerbestrijding, no. 10817). JFMJ and TML have obtained a research grant from Sebia.

## DISCLOSURES

The authors have no conflicts of interest to disclose.

## Supplementary Material


