# Supplementary material for: Retrospective Longitudinal Monitoring of Multiple Myeloma Patients by Mass Spectrometry Using Archived Serum Protein Electrophoresis Gels and De Novo Sequence Analysis
Source: Hemasphere. 2022 Aug 2;6(8):e758. doi: 10.1097/HS9.0000000000000758 (PMC9348860; doi:10.1097/HS9.0000000000000758)

Retrospective longitudinal monitoring of multiple myeloma patients by mass spectrometry using archived serum protein electrophoresis gels and de novo sequence analysis

Supplemental Digital Content

## Supplemental Methods

### Patient material

Materials used in this study are diagnostic SPEP gels (Hydragel, Sebia) from MM patients. The gels in this study were archived at the Department of Clinical Chemistry, Erasmus MC, but storage of gels is also common at the other author institution. For SPEP analysis, MM patient serum has been processed and used for diagnostic purposes. The archived SPEP gels were stored as dried agarose gels supported by a plastic carrier at room temperature. MM patients (n=9) were retrospectively selected based on the data in the hospital information system. Patients were selected to have SPEP detectable M-protein at diagnosis (>1 g/L), and at least one period when the M-protein was absent by SPEP and IFE, followed by again detectable M-protein. From these patients M-protein DNA/RNA sequence was not available. In the Supplemental Digital Content the available clinical data has been listed for the selected patients including SPEP, free light chain, urine electrophoresis, bone marrow MRD data (10-4 flow cytometry) as well as the main treatments. Patient samples and clinical data were coded as specified in the Dutch code of conduct for biomedical research (institutional review board approval MEC-2019-0342). The SPEP gels were generated for diagnostic purposes from 2010 until 2016, and all gel samples that were available for these patients have been digested and measured by MS. Table S1 describes patient and sample characteristics and supplemental Table S2 provides clinical data and information about patient treatment.

A dilution series was prepared from a sample (16.6 g/L of M-protein by SPEP) of a patient that also has DNA sequence data on the M-protein heavy chain 11,13. The dilutions were analyzed using SPEP to validate the de novo sequencing approach and the quantitative performance of our method.

### Excision and trypsin digestion of the M-protein bands

Cutting the M-protein bands was performed in steps to ensure that the correct area of the gamma/beta fraction was cut even when the M-protein band was not visible. The width of the M-protein band and the distance to the albumin band were measured. At time points when M-protein was not detectable by SPEP, these metrics were used to locate the proper position in the gel. With a scalpel the M-protein band was excised from the gels. The dilution series of the reference patient was cut as described previously.

Each M-protein band was digested separately in a 1.5 mL Eppendorf tube, all volumes were 60 µL unless specified otherwise. First, the bands were washed with water, 50% acetonitrile (ACN), 100% ACN and 50% ACN in 100 mM ammonium bicarbonate (ABC), then dried in Savant SC210A SpeedVac concentrator (Thermo Fisher Scientific, Munich, Germany) for 5 minutes. Reduction and alkylation were performed using 10 mM dithiothreitol at 56°C for 45 minutes, and 55 mM iodoacetamide at room temperature in the dark for 30 minutes. Afterwards, gel bands were washed with water, 50% ACN, 100% ACN and 50% ACN in 100 mM ABC and dried. Then, 0.1 % RapiGest SF (Waters, Milford, MA) in 50 mM ABC was added for 10 minutes at 37 °C. After drying, 600 ng of gold-grade trypsin (Promega, Madison, WI) in 50 mM ABC was added and incubated at 4°C for 5 minutes. The solution was collected and 50 mM ABC was added, followed by incubation at 37°C overnight. Tryptic peptides were extracted once with 1% trifluoroacetic acid (TFA) and twice with 0.1% TFA in 50% ACN. All extracts and trypsin solution were combined and completely dried before resuspension in 25 µL 0.1% TFA. All samples were cleaned with C18 ZipTips (Millipore, Burlington, MA).

### Liquid chromatography-mass spectrometry (LC-MS)

Liquid chromatography was carried out on a nano-LC system (Ultimate 3000, Thermo Fisher Scientific, Munich, Germany). Sample volumes of 10 µL were injected and separated on a C18 column (Acclaim PepMap, 75 µm ID × 250 mm, 2 µm, 100 Å ; Thermo Fisher Scientific, Munich, Germany). A 30 minute gradient of 4%–38% was used; A: 0.1% formic acid in water; B: 80% acetonitrile, 0.08% formic acid in water.

### DDA MS measurements

Data Dependent Acquisition (DDA, Shotgun) measurements were performed on a QExactive HF Orbitrap mass spectrometer (Thermo Fisher Scientific, Bremen, Germany). MS1 scans (375.00–1500.00 m/z) had a resolution of 60,000, AGC target 3e6, maximum injection time 60 ms. MS/MS spectra used HCD fragmentation at 28% normalized collision energy on the top 20 precursors at resolution 15,000, dynamic exclusion 40 s, AGC target 5e5, maximum injection time 50 ms and isolation width 1.4 m/z. The MS data have been deposited to repository with Proteome Exchange accession PXD034406, and is available at <https://panoramaweb.org/MSMRDGelStudy.url> .

### Targeted MS measurements

Targeted MS/MS spectra used HCD fragmentation at 27% normalized collision energy, resolution 30,000, AGC target 5e5, maximum injection time 250 ms and isolation width 1.4 m/z. All peptides are listed in Supplemental Table S-2 as used for quantitation.

### *De novo* sequencing of the mass spectra

*De novo* sequencing was performed on DDA as well as targeted MS raw data files using Peaks Studio 6.0 (Bioinformatics Solutions Inc., Waterloo, Canada). The following settings were used: enzyme trypsin, Orbitrap instrument, HCD fragmentation, methionine oxidation and carbamidomethylation were selected as variable and fixed post-translational modifications, respectively. Parent ion tolerance of 5 ppm and fragment ion tolerance of 0.02 Da were allowed. Where targeted and DDA MS data resulted in de novo sequences with a difference, those based on the targeted data were used due to the superior signal in these.

### Selection of patient-specific M-protein peptides from *de novo* sequencing data

Of each patient, the sample of the first timepoint was measured in order to select patient-specific M-protein peptides. Peptide candidates had to fulfill several criteria. First, a patient-specific M-protein peptide candidate has to be highly abundant in the patient of interest and absent or a minimal signal (<1%) in all other patient samples. Progenesis QI (version 2.0, Waters, Milford, MA) was used for label-free quantitation of MS data and combined with de novo sequencing information. Other selection criteria were: the peptide has an de novo sequencing total local confidence (TLC) above 7; the peptide should not be identical to an immunoglobulin germline sequence (IMGT), ensuring that the sequence contains mutations that can make it specific to the patient. Also, a search with IgBlast (www.ncbi.nlm.nih.gov/igblast/) was used to show peptide homology to immunoglobulins, which was >68% for all selected peptides.

### Methionine oxidation

During *de novo* sequencing, oxidation of methionine residues was allowed as a modification in the software. Indeed, oxidized methionine residues were frequently observed in the data, and often the oxidized form of the peptide also gave a stronger signal than the native peptide. This suggests that oxidation events may be common in SPEP gels during extended storage. As stronger signals were observed, such oxidized peptides were also selected as monitoring targets. In such cases, the native peptide was also included in the analysis which showed that such peptide modification variants did not give a discrepant view of disease dynamics, suggesting that oxidation does not interfere with conclusions in current study (Figure S-3). Furthermore, it was found that peptides derived from the heavy and light chains performed similarly for quantitation by mass spectrometry, as shown for Patient 2 and also for the dilution series.

### Longitudinal M-protein monitoring

Patient-specific M-protein peptides were used to monitor the disease course of each patient. All samples were measured with targeted MS data analyzed in Skyline. A spectrum library was created based on data from the initial sample, and subsequent samples with a library dot product exceeding 0.8 were considered positive. The area of the peaks were plotted with other clinical information available for these patients. As the clinical records were inconsistent in reporting SPEP data below 5 g/L numerically, all numeric data between 1 and 5 g/L were plotted as 5 g/L. Samples shown as negative for M-protein were assessed as such by a laboratory specialist based on the combined data from SPEP, IFE and nephelometry that was available at the original time of analysis.

**Table S1.** Patient characteristics

| **Patient number** | **M-protein** | **SPEP fraction** | **Days follow-up**^a^ | **Samples measured with MS**^b^ |
| --- | --- | --- | --- | --- |
| 1 | IgA lambda | beta | 1422 | 33 |
| 2 | IgG kappa | gamma | 584 | 12 |
| 3 | IgG kappa | gamma | 760 | 31 |
| 4 | IgA lambda | gamma | 1512 | 30 |
| 5 | IgG kappa | gamma | 1707 | 36 |
| 6 | IgA kappa | gamma | 702 | 26 |
| 7 | IgG kappa | gamma | 918 | 17 |
| 8 | IgG kappa | gamma | 1799 | 32 |
| 9 | IgG kappa | gamma | 1914 | 28 |

^a^number of days between the first and the last time point in the disease, measured by mass spectrometry (MS)

^b^each sample represents serum of a distinct time point in the disease course, analyzed with serum protein electrophoresis (SPEP) and excised from the gel for MS measurement, as described in the method section

**Table S2** - Clinical information Patient 1-9.

Clinical data obtained from routine tests during the course of disease. Indicated is the sampling day relative to the first sample available for this study, serum protein electrophoresis result (SPEP), free light chain data for kappa and lambda light chains by nephelometry as well as their ratio, urine protein electrophoresis (UPEP) results, the outcome of bone marrow evaluation with flow cytometry and a summary of the main treatment events.

Patient 1

| **Day** | **SPEP (M-protein g/L)** | **Free light chain kappa (mg/L)** | **Free light chain lambda (mg/L)** | **K/L ratio** | **UPEP g/24h** | **Bone marrow** | **Treatments** |
| --- | --- | --- | --- | --- | --- | --- | --- |
| 0 | 20 |  |  |  |  |  | Lenalidomide |
| 29 | 19 | <6,60 | 1446.00 | <0,01 | 3.80 |  |  |
| 48 | 18 |  |  |  |  |  |  |
| 57 |  |  |  |  |  |  | Lenalidomide discontinued |
| 62 | 19 | <6,85 | 1200.00 | <0,01 | 4.74 |  |  |
| 99 | 22 | <6,00 | 932.00 | <0,01 | 4.47 |  |  |
| 153 | 22 | <6,00 | 1136.00 | <0,01 |  |  |  |
| 164 | Negative |  |  |  |  |  |  |
| 176 |  |  |  |  |  | 30% of bonemarrow leukocytes |  |
| 178 |  |  |  |  |  |  | Bortezomib, dexamethasone |
| 201 | 13 | <6,00 | 15.90 | <0,38 |  |  |  |
| 229 | 9 |  |  |  |  |  |  |
| 245 | 9 | <6,00 | 6.65 | NQ |  |  |  |
| 256 | 9 |  |  |  |  |  |  |
| 264 | Negative |  |  |  |  |  |  |
| 293 | Negative |  |  |  |  |  |  |
| 331 |  |  |  |  |  |  | Treatment stopped --> CR |
| 335 | Negative |  |  |  |  |  |  |
| 424 | Negative | 7.95 | 11.40 | 0.70 |  |  |  |
| 487 | Negative | 6.28 | 16.10 | 0.39 |  |  |  |
| 579 | 11 |  |  |  |  |  |  |
| 656 | 13 |  |  |  |  |  |  |
| 697 | 18 | <6,30 | 955.00 | <0,01 |  |  |  |
| 718 | 19 | <6,30 | 1062.00 | <0,01 | 2.74 |  |  |
| 784 | 26 | <6.30 | 2200 | <0.01 |  |  |  |

Patient 1 (continued)

| 816 |  |  |  |  |  | monoclonal plasma cell population present | Bortezomib, cyclophosphamide, dexamethasone (VCD) |
| --- | --- | --- | --- | --- | --- | --- | --- |
| 883 | 8 | <6.60 | <4.90 | NQ |  |  |  |
| 904 | 8 | <6,60 | <4,90 | NQ |  |  |  |
| 909 | 8 | <6,60 | <4,90 | NQ |  |  |  |
| 969 |  |  |  |  |  |  | Treatment stopped --> sCR |
| 973 |  |  |  |  |  | no monoclonal plasma cell population |  |
| 1000 | Negative | <6.60 | <4.50 | NQ |  |  |  |
| 1021 | Negative |  |  |  |  |  |  |
| 1072 | Negative |  |  |  |  |  |  |
| 1131 | Negative | 9.16 | 26.40 | 0.35 |  |  |  |
| 1170 | Negative |  |  |  |  |  |  |
| 1320 | 8 |  |  |  |  |  |  |
| 1359 | 8 |  |  |  |  |  |  |
| 1387 | 9 | 4.69 | 746.00 | <0,01 |  |  |  |
| 1422 | 10 | <4.00 | 464.00 | NQ |  |  |  |
| 1456 |  |  |  |  |  |  | Lenalidomide, cyclophosphamide, prednisone |

Patient 2

| **Day** | **SPEP (M-protein g/L)** | **Free light chain kappa (mg/L)** | **Free light chain lambda (mg/L)** | **K/L ratio** | **UPEP g/24h** | **Bone marrow** | **Treatments** |
| --- | --- | --- | --- | --- | --- | --- | --- |
| 0 | 18 | 6.71 | 5.83 | 1.15 |  | monoclonal plasma cell population present | VCD |
| 33 |  |  |  |  |  |  | Treatment stopped --> VGPR |
| 41 | 5 | <6,00 | 7.83 | NQ |  | no monoclonal plasma cell population |  |
| 94 |  |  |  |  |  |  | HDM + ASCT |
| 153 | 5 |  |  |  |  |  |  |
| 155 |  |  |  |  |  |  | Lenalidomide maintenance |
| 260 | 5 | 21.80 | 26.60 | 0.82 |  |  |  |
| 315 | Negative | 25.50 | 27.50 | 0.93 |  |  |  |
| 329 |  |  |  |  |  | no monoclonal plasma cell population |  |
| 400 | Negative | 15.70 | 22.00 | 0.71 |  | no monoclonal plasma cell population |  |
| 482 | Negative | 12.80 | 19.10 | 0.67 |  |  |  |
| 511 | 5 | 16.20 | 18.50 | 0.88 | Negative | |  |
| 518 | 5 |  |  |  |  |  |  |
| 539 | 6 | 22.80 | 15.70 | 1.45 |  |  |  |
| 563 | 11 | 46.70 | 12.80 | 3.65 |  |  |  |
| 584 | 19 | 275.0 | 10.40 | 26 |  |  |  |
| 591 |  |  |  |  |  | 0.02% monoclonal plasmacells |  |
| 610 |  |  |  |  |  |  | Lenalidomide, cyclophosphamide, prednisone |

Patient 3

| **Day** | **SPEP (M-protein g/L)** | **Free light chain kappa (mg/L)** | **Free light chain lambda (mg/L)** | **K/L ratio** | **UPEP g/24h** | **Bone marrow** | **Treatments** |
| --- | --- | --- | --- | --- | --- | --- | --- |
| 0 | 17 |  |  |  |  |  |  |
| 21 | 20 |  |  |  |  |  |  |
| 63 | 10 |  |  |  |  |  |  |
| 88 | 5 |  |  |  |  |  |  |
| 119 |  |  |  |  |  |  | Carfilzomib, lenalidomide, dexamethasone |
| 120 | 5 |  |  |  |  |  |  |
| 144 | 5 | <6,60 | <4,70 | NQ |  |  |  |
| 176 | 5 |  |  |  |  |  |  |
| 203 | 5 |  |  |  |  |  |  |
| 233 | 5 | <6,00 | 7.05 | NQ |  |  |  |
| 259 | Negative | <6,00 | 8.30 | NQ |  |  |  |
| 289 | Negative |  |  |  |  |  |  |
| 315 | Negative |  |  |  |  |  |  |
| 329 | Negative |  |  |  |  |  |  |
| 343 | Negative |  |  |  |  |  |  |
| 357 | Negative |  |  |  |  |  |  |
| 392 | Negative |  |  |  |  |  |  |
| 406 | Negative |  |  |  |  |  |  |
| 435 | 5 |  |  |  |  |  |  |
| 459 | 5 |  |  |  |  |  |  |
| 514 | 5 |  |  |  |  |  |  |
| 549 | 5 |  |  |  |  |  |  |
| 571 | 5 |  |  |  |  |  |  |
| 602 | 5 |  |  |  |  |  |  |
| 632 | 5 |  |  |  |  |  |  |
| 660 | 5 |  |  |  |  |  |  |
| 687 | 5 |  |  |  |  |  |  |
| 714 | 10 | 14.10 | 5.75 | 2.45 |  |  |  |
| 730 | 9 | 15.00 | <5,20 | NQ |  |  |  |
| 738 | 10 | 18.50 | <4,90 | NQ |  |  |  |
| 749 | 6 |  |  |  |  | 3% monoclonal plasmacells |  |
| 760 | 15 | 33.50 | 5.46 | 6.14 |  |  |  |
| 819 |  |  |  |  |  |  | Lenalidomide, prednisone, cyclophosphamide |
| 1031 |  |  |  |  |  |  | Treatment stopped |

Patient 4

| **Day** | **SPEP (M-protein g/L)** | **Free light chain kappa (mg/L)** | **Free light chain lambda (mg/L)** | **K/L ratio** | **UPEP g/24h** | **Bone marrow** | **Treatments** |
| --- | --- | --- | --- | --- | --- | --- | --- |
| 0 | 5 | 6.40 | 30.20 | 0.21 |  |  | Carfilzomib, thalidomide, dexamethasone (Carthadex) |
| 13 | 5 | 8.65 | 27.00 | 0.32 | Negative | |  |
| 41 | 5 | 6.64 | 12.40 | 0.54 |  |  |  |
| 65 | 5 | 5.69 | 10.30 | 0.55 |  |  |  |
| 90 |  |  |  |  |  |  | Treatment stopped --> VGPR |
| 114 | 5 | <5,40 | 5.97 | NQ | Negative | |  |
| 149 |  |  |  |  |  |  | HDM + ASCT |
| 204 | 5 | 16.40 | 12.20 | 1.34 | Negative | |  |
| 241 |  |  |  |  |  |  | Carthadex consolidation |
| 252 | Negative |  |  |  | Negative | |  |
| 262 | Negative | <5,40 | <5,20 | NQ |  |  |  |
| 279 | Negative |  |  |  |  |  |  |
| 303 | Negative |  |  |  |  |  |  |
| 321 | Negative |  |  |  |  |  |  |
| 333 |  |  |  |  |  |  | Consolidation stopped |
| 391 | Negative | 8.89 | 14.40 | 0.62 | Negative | |  |
| 468 | Negative | 9.31 | 17.50 | 0.53 | Negative | |  |
| 521 | Negative | <6.60 | 15.40 | NQ |  |  |  |
| 577 | Negative |  |  |  | Negative | |  |
| 625 | Negative | 6.95 | 11.40 | 0.61 | Negative | |  |
| 688 | Negative | 7.69 | 10.40 | 0.74 | Negative | |  |
| 745 | Negative | 5.86 | 18.00 | 0.33 | Negative | |  |
| 827 | Negative | 8.77 | 14.80 | 0.59 | Negative | |  |
| 892 | Negative | 9.22 | 19.10 | 0.48 | Negative | |  |
| 958 | Negative | 5.77 | 11.80 | 0.49 | Negative | |  |
| 1016 | Negative | 7.75 | 19.10 | 0.41 | Negative | |  |
| 1093 | Negative | 6.08 | 16.60 | 0.37 | Negative | |  |
| 1165 | Negative | 8.76 | 19.80 | 0.44 | Negative | |  |
| 1219 | Negative | 10.60 | 19.60 | 0.54 | Negative | |  |
| 1289 | Negative | 11.70 | 19.90 | 0.59 | Negative | |  |
| 1373 | 5 | 13.40 | 22.80 | 0.59 | Negative | |  |
| 1437 | 5 | 20.20 | 24.70 | 0.82 | Negative | |  |
| 1465 | 5 | 21.40 | 25.70 | 0.83 | Negative | |  |
| 1512 | 5 | 21.10 | 25.30 | 0.83 | Negative | |  |

Patient 5

| **Day** | **SPEP (M-protein g/L)** | **Free light chain kappa (mg/L)** | **Free light chain lambda (mg/L)** | **K/L ratio** | **UPEP g/24h** | **Bone marrow** | **Treatments** |
| --- | --- | --- | --- | --- | --- | --- | --- |
| 0 | 5 | 5.46 | 5.79 | 0.94 |  |  | Carthadex |
| 15 | 5 | 5.78 | 8.78 | 0.66 |  |  |  |
| 38 |  |  |  |  |  |  | Treatment stopped --> VGPR |
| 46 | 5 | <6,60 | 6.10 | NQ | Negative | |  |
| 60 | 5 | 6.98 | 7.05 | 0.99 |  |  |  |
| 76 | 5 | 10.30 | 9.41 | 1.10 |  |  |  |
| 99 |  |  |  |  |  |  | HDM + ASCT, after Carthadex consolidation |
| 155 | 5 |  |  |  |  |  |  |
| 186 | Negative | <6,00 | 4.55 | NQ |  |  |  |
| 196 | Negative |  |  |  |  |  |  |
| 211 | Negative | <6,00 | 4.96 | NQ | Negative | |  |
| 238 | Negative | <6,00 | 4.59 | NQ |  |  |  |
| 277 | Negative |  |  |  |  |  |  |
| 340 | Negative |  |  |  |  |  |  |
| 400 | 5 |  |  |  | Negative | |  |
| 470 | 5 | 8.19 | 9.12 | 0.90 | Negative | |  |
| 497 | Negative | 12.00 | 11.40 | 1.05 |  |  |  |
| 543 | Negative | 12.60 | 24.80 | 0.51 | Negative | |  |
| 588 | Negative | 10.10 | 15.30 | 0.66 | Negative | |  |
| 635 | Negative |  |  |  |  |  |  |
| 673 | Negative |  |  |  |  |  |  |
| 732 | Negative |  |  |  |  |  |  |
| 792 | Negative |  |  |  | Negative | |  |
| 866 | Negative |  |  |  | Negative | |  |
| 925 | Negative |  |  |  | Negative | |  |
| 981 | Negative |  |  |  | Negative | |  |
| 1036 | Negative |  |  |  | Negative | |  |
| 1100 | Negative |  |  |  |  |  |  |
| 1159 | Negative |  |  |  | Negative | |  |
| 1225 | Negative |  |  |  | Negative | |  |
| 1288 | Negative |  |  |  | Negative | |  |
| 1446 | 5 |  |  |  | Negative | |  |
| 1519 | 5 |  |  |  |  |  |  |
| 1546 | 5 |  |  |  | Negative | |  |
| 1581 | 5 |  |  |  | Negative | |  |
| 1651 | 5 |  |  |  |  |  |  |
| 1679 | 5 |  |  |  | Negative | |  |
| 1707 | 5 |  |  |  |  |  |  |

Patient 6

| **Day** | **SPEP (M-protein g/L)** | **Free light chain kappa (mg/L)** | **Free light chain lambda (mg/L)** | **K/L ratio** | **UPEP g/24h** | **Bone marrow** | **Treatments** |
| --- | --- | --- | --- | --- | --- | --- | --- |
| 0 | 26 |  |  |  |  |  |  |
| 37 | 11 |  |  |  |  |  |  |
| 65 | 7 |  |  |  |  |  |  |
| 97 | 5 | 394.00 | 4.60 | 85.65 |  |  |  |
| 118 |  |  |  |  |  |  | Lenalidomide and dexamethasone |
| 135 | 6 |  |  |  |  |  |  |
| 166 | 5 |  |  |  |  |  |  |
| 173 | 5 |  |  |  |  |  |  |
| 180 | 5 |  |  |  |  |  |  |
| 191 | 5 | 47.80 | 4.64 | 10.30 |  |  |  |
| 219 | 5 |  |  |  |  |  |  |
| 233 | 5 | 33.10 | 4.15 | 7.98 |  |  |  |
| 250 | 5 | 22.40 | <3,90 | NQ |  |  |  |
| 275 | 5 | 18.10 | 7.67 | 2.36 |  |  |  |
| 288 | 5 | 15.80 | <5,20 | NQ |  |  |  |
| 306 | 5 | 12.50 | <5,20 | NQ |  |  |  |
| 334 | 5 | 10.40 | <5,20 | NQ |  |  |  |
| 358 | 5 | 7.39 | <5,20 | NQ |  |  |  |
| 387 | 5 | 8.50 | <5,20 | NQ |  |  |  |
| 420 | Negative |  |  |  |  |  |  |
| 422 |  |  |  |  |  |  | Treatment stopped |
| 450 | Negative |  |  |  |  |  |  |
| 474 | Negative | 10.50 | <5,20 | NQ |  |  |  |
| 488 | Negative | 11.50 | <5,20 | NQ |  | 0.06% monoclonal plasmacells (CD38+/CD19-/CD45-/CyIgKappa+/CD56+), 0.02% normal plasmacells. |  |
| 548 | 5 | 22.50 | <5,20 | NQ |  |  |  |
| 611 | 5 | 33.30 | 6.29 | 5.29 |  |  |  |
| 671 | 5 | 170.00 | 5.55 | 30.63 |  |  |  |
| 702 | 5 |  |  |  |  |  |  |

Patient 7

| **Day** | **SPEP (M-protein g/L)** | **Free light chain kappa (mg/L)** | **Free light chain lambda (mg/L)** | **K/L ratio** | **UPEP g/24h** | **Bone marrow** | **Treatments** |
| --- | --- | --- | --- | --- | --- | --- | --- |
| 0 | 29 |  |  |  |  |  | Bortezomib and dexamethasone |
| 15 | 14 |  |  |  |  |  |  |
| 19 | 13 |  |  |  |  |  |  |
| 48 | 5 |  |  |  |  |  |  |
| 72 | 5 |  |  |  |  |  |  |
| 91 | 5 |  |  |  |  |  |  |
| 105 | Negative | 13.00 | <4,70 | NQ |  |  |  |
| 112 | Negative |  |  |  |  | no monoclonal plasma cell population |  |
| 215 |  |  |  |  |  |  | Treatment stopped --> sCR |
| 296 | Negative |  |  |  |  |  |  |
| 344 | Negative |  |  |  |  | no monoclonal plasma cell population |  |
| 426 | Negative |  |  |  |  |  |  |
| 511 | Negative |  |  |  |  |  |  |
| 853 | 25 |  |  |  |  |  |  |
| 884 |  |  |  |  |  |  | Bortezomib, lenalidomide, prednisone |
| 890 | 29 |  |  |  |  |  |  |
| 895 | 28 |  |  |  |  |  |  |
| 897 | 24 |  |  |  |  |  |  |
| 918 | 17 |  |  |  |  |  |  |

Patient 8

| **Day** | **SPEP (M-protein g/L)** | **Free light chain kappa (mg/L)** | **Free light chain lambda (mg/L)** | **K/L ratio** | **UPEP g/24h** | **Bone marrow** | **Treatments** |
| --- | --- | --- | --- | --- | --- | --- | --- |
| 0 | 5 |  |  |  |  |  | Dasatinib, dexamethasone, lenalidomide |
| 27 | 5 |  |  |  |  |  |  |
| 56 | 5 |  |  |  |  |  |  |
| 99 | 5 |  |  |  |  |  |  |
| 154 | 5 |  |  |  |  |  |  |
| 210 | 5 |  |  |  |  |  |  |
| 238 | 5 |  |  |  |  |  |  |
| 281 | 5 |  |  |  |  |  |  |
| 309 | 5 |  |  |  |  |  |  |
| 337 | 5 |  |  |  |  |  |  |
| 364 | 5 |  |  |  |  |  |  |
| 396 | 5 |  |  |  |  |  |  |
| 420 | 5 |  |  |  |  |  |  |
| 434 | Negative |  |  |  |  |  |  |
| 448 | Negative |  |  |  |  |  |  |
| 463 | Negative |  |  |  |  |  |  |
| 481 | Negative |  |  |  |  |  |  |
| 491 | Negative |  |  |  |  |  |  |
| 523 | 5 |  |  |  |  |  |  |
| 533 | 5 |  |  |  |  |  |  |
| 557 | Negative |  |  |  |  |  |  |
| 558 |  |  |  |  |  |  | Treatment stopped --> VGPR |
| 567 | Negative |  |  |  |  |  |  |
| 596 | 5 |  |  |  |  |  |  |
| 641 | 5 |  |  |  |  |  |  |
| 673 | 5 |  |  |  |  |  |  |
| 736 | 5 |  |  |  |  |  |  |
| 909 | 5 |  |  |  |  |  |  |
| 1035 | 5 |  |  |  |  |  |  |
| 1155 | 5 |  |  |  |  |  |  |
| 1434 | 5 |  |  |  |  |  |  |
| 1547 | 5 |  |  |  |  |  |  |
| 1799 | 5 |  |  |  |  |  |  |

Patient 9

| **Day** | **SPEP (M-protein g/L)** | **Free light chain kappa (mg/L)** | **Free light chain lambda (mg/L)** | **K/L ratio** | **UPEP g/24h** | **Bone marrow** | **Treatments** |
| --- | --- | --- | --- | --- | --- | --- | --- |
| 0 | 15 |  |  |  |  |  |  |
| 87 | 17 |  |  |  |  |  |  |
| 108 | 18 |  |  |  |  |  |  |
| 231 | 24 |  |  |  |  |  |  |
| 298 | 22 |  |  |  |  |  |  |
| 389 | 25 |  |  |  |  |  |  |
| 406 | 28 |  |  |  |  | 9.2% of bone marrow leukocytes |  |
| 437 | 29 |  |  |  |  |  |  |
| 451 | 27 |  |  |  |  |  |  |
| 462 | 11 |  |  |  |  |  |  |
| 496 |  |  |  |  |  |  | Panabinostat, bortezomib, dexamethasone |
| 508 | 5 |  |  |  |  |  |  |
| 546 | 5 |  |  |  |  |  |  |
| 558 | 5 |  |  |  |  |  |  |
| 567 | 5 |  |  |  |  |  |  |
| 595 | 5 |  |  |  |  |  |  |
| 618 |  |  |  |  |  |  | Treatment stopped --> VGPR |
| 620 | 5 |  |  |  |  |  |  |
| 733 | 5 |  |  |  |  |  |  |
| 847 | Negative |  |  |  |  |  |  |
| 966 | Negative |  |  |  |  |  |  |
| 990 |  |  |  |  |  | 0.9% of bonemarrow leukocytes |  |
| 1159 | Negative |  |  |  |  |  |  |
| 1263 | Negative |  |  |  |  |  |  |
| 1382 | Negative |  |  |  |  |  |  |
| 1505 | 5 |  |  |  |  |  |  |
| 1631 | 5 |  |  |  |  |  |  |
| 1705 | 5 |  |  |  |  |  |  |
| 1769 | 5 |  |  |  |  |  |  |
| 1823 | 5 |  |  |  |  |  |  |
| 1914 | 5 |  |  |  |  |  |  |

**Table S3.** *De novo* sequence results for clonotypic peptides of the multiple myeloma patients

| **Patient number** | **M-protein chain** | ***De novo* M-protein peptide sequence** | **ALC**% ^c^ | **Germline peptide sequence** |
| --- | --- | --- | --- | --- |
| 1 | heavy | DSVFLQMNSLRb | 73 | NSLYLQMNSLR |
| 2 | heavy | DGQLVESGGGSAQPNSLR ^a^ | 86 | EDQLVESGGGLVQPGGSLR |
| 3 | heavy | LSCEASGFTFR | 91 | LSCAASGFTF |
| 4 | heavy | SPPVSVSHVEANSPGQTASLTCSGDK | 72 | SYELTQLPSVSVSPGQTARITCSGDV |
| 5 | heavy | MTNMDPVDTATYYCAR ^b^ | 76 | MTNMDPVDTATYYCAR ^d^ |
|  | light | LVLTQSPATLSLSASPNAAK ^a^ | 73 | IVLTQSPATLSLSP |
| 6 | heavy | VELLVESGGDLVQPGGSLR | 82 | LVESGGGLVQPGGSLR |
|  | light | LEVLTQSPGTLSLSPDAR | 82 | VLTQSPGTLSLSPGER |
| 7 | heavy | EDTALFYCVK | 84 | EDTAVYYCVK |
|  | light | DLQMTQSPSSLSASVGDK ^b^ | 78 | DIQMTQSPSSLSASVGDR |
| 8 | heavy | SMTAADTGVYYCAR | 80 | SVTAVDTGVYYCAR |
|  | light | QLDLTQSPSSLSASVGDR | 72 | IQLTQSPSSLSASVGDR |
| 9 | light | LSLYGASNLQGGVPSK ^a^ | 76 | LLIYSASNLQSGVPSR |

^a^*de novo* sequences were further refined based on targeted MS/MS data before use in quantitation

^b^ The peptide was monitored with all methionine residues in the oxidized form

^c^ALC = average local confidence score from PEAKS

^d^ The germline peptide differs by lacking an N-terminal tryptic site.

**Table S4**. Sequence, *m/z*, charge states and fragment types of selected patient-specific M-protein peptides used for M-protein monitoring with targeted mass spectrometry.

| **Patient** | **Sequence** | **m/z** | **z** | **Fragments** |
| --- | --- | --- | --- | --- |
| 1 | DSVFLQM[Ox]NSLR | 663.3295 | 2 | y5-8 |
| 2 | DGQLVESGGGSAQPGGSLR | 886.4321 | 2 | y6; y11-15 |
| 3 | LSC[CAM]EASGFTFR | 637.7953 | 2 | y5-10 |
| 4 | SPPVSVSHVEANSPGQTASLTC[CAM]SGDK | 871.4134 | 3 | y6; y8-10; y13 |
| 5 | M[Ox]TNM[Ox]DPVDTATYYC[CAM]AR | 970.8948 | 2 | y4; y6-9; y11 |
| 6 | VELLVESGGDLVQPGGSLR | 963.0206 | 2 | y6-9; y11-14 |
| 7 | EDTALFYC[CAM]VK | 623.2946 | 2 | y4-8 |
| 8 | SM[Ox]TAADTGVYYC[CAM]AR | 791.3372 | 2 | y5; y7-12 |
| 9 | LNSAGYLSLQGGVPSK | 795.9279 | 2 | y2-y8 |

[Ox] = oxidation

[CAM] = carbamidomethylation

**Table S5** DNA and *de novo* sequencing results of the reference patient

Differences between the patient specific M-protein and the most homologous germline sequence are printed bold. Differences between the *de novo* sequencing result and the DNA-derived sequence are in red. No light chain DNA data was available to validate sequences obtained from *de novo* sequencing for the light chain. Trypsin cleavage sites are indicated with | symbols.


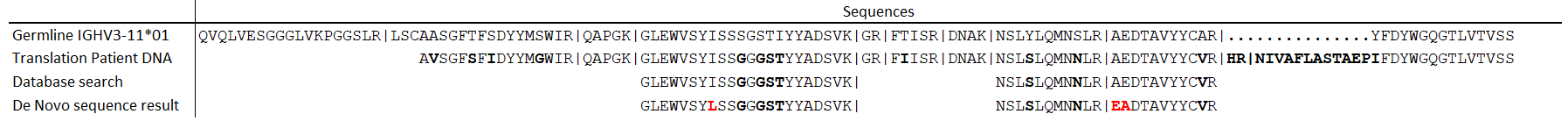


**Figure S1**. Mass spectra and *de novo* sequence analysis based on DDA data acquired for the patients. These correspond to the peptides listed in Table 2. Data for Patients 1-9 is shown in panels A-I, respectively. The analysis for the DNA-verified reference patient is shown in panel J.

1. Patient 1 Heavy chain


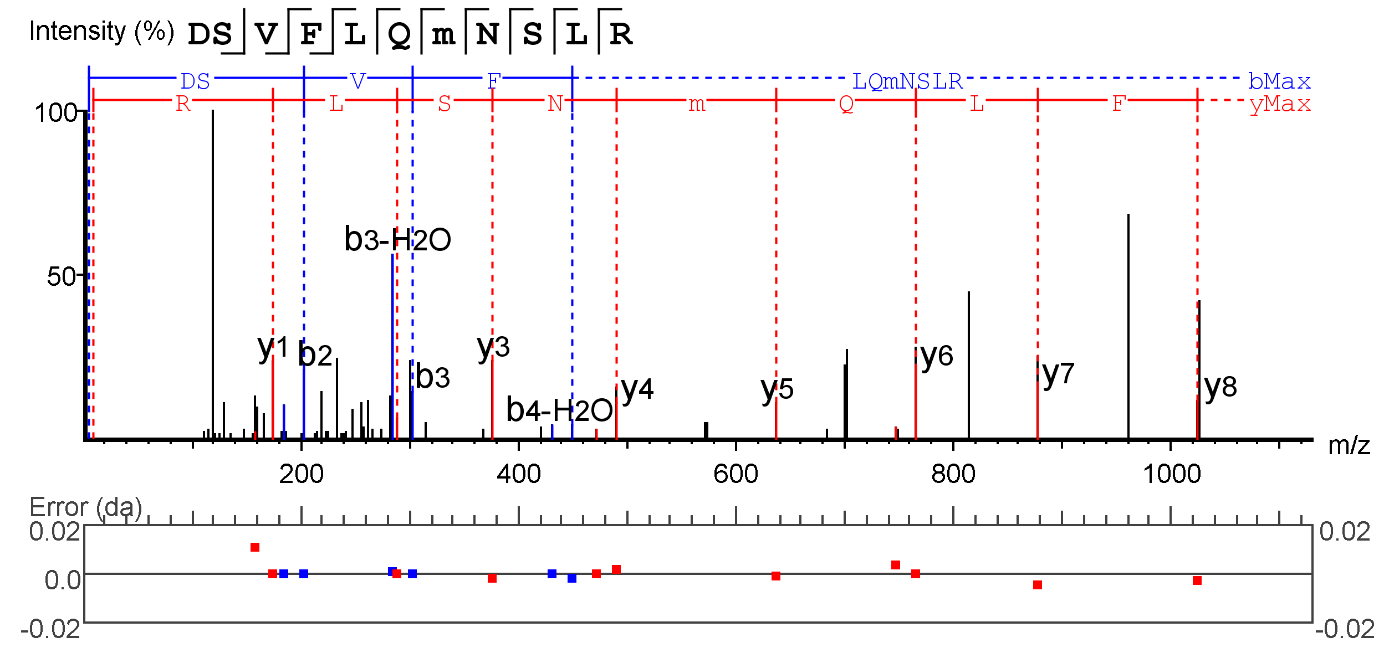


1. Patient 2 Heavy chain


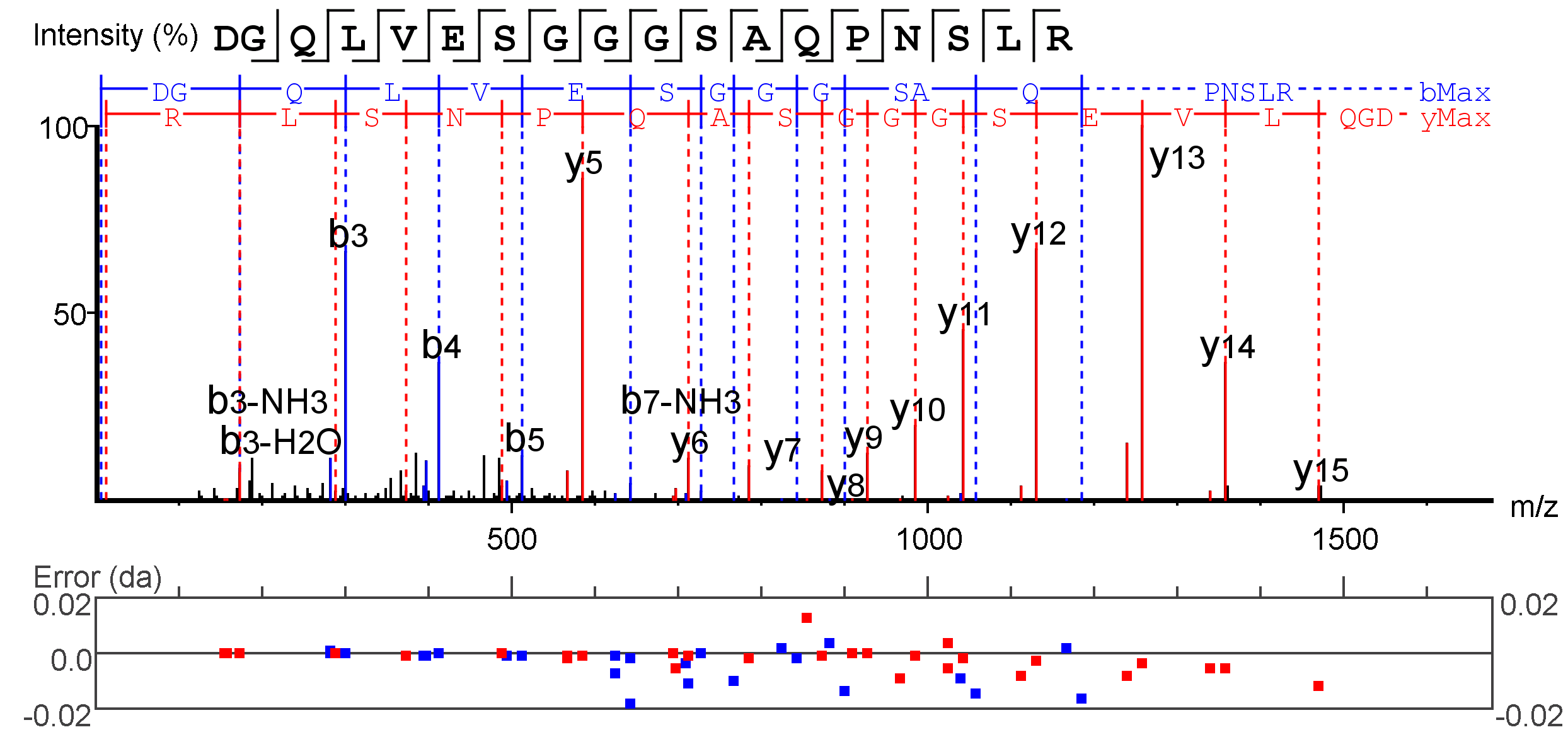


1. Patient 3 - Heavy chain


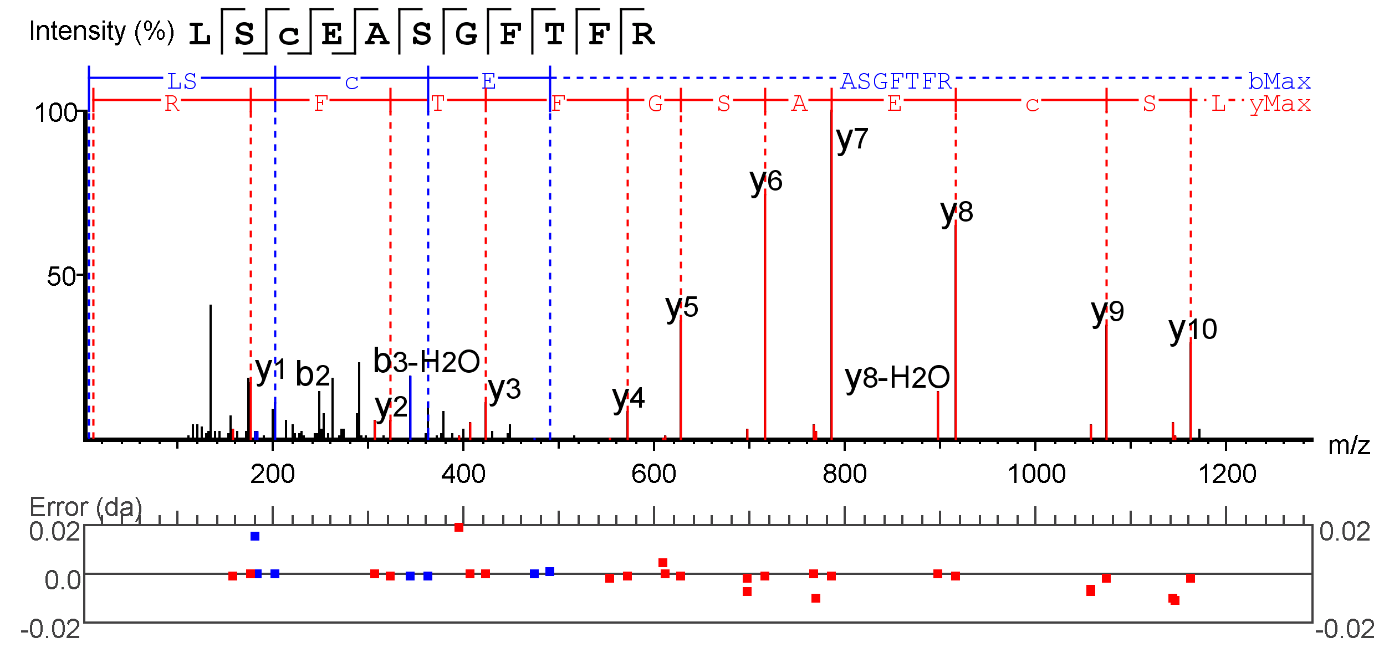


1. Patient 4 - Heavy chain


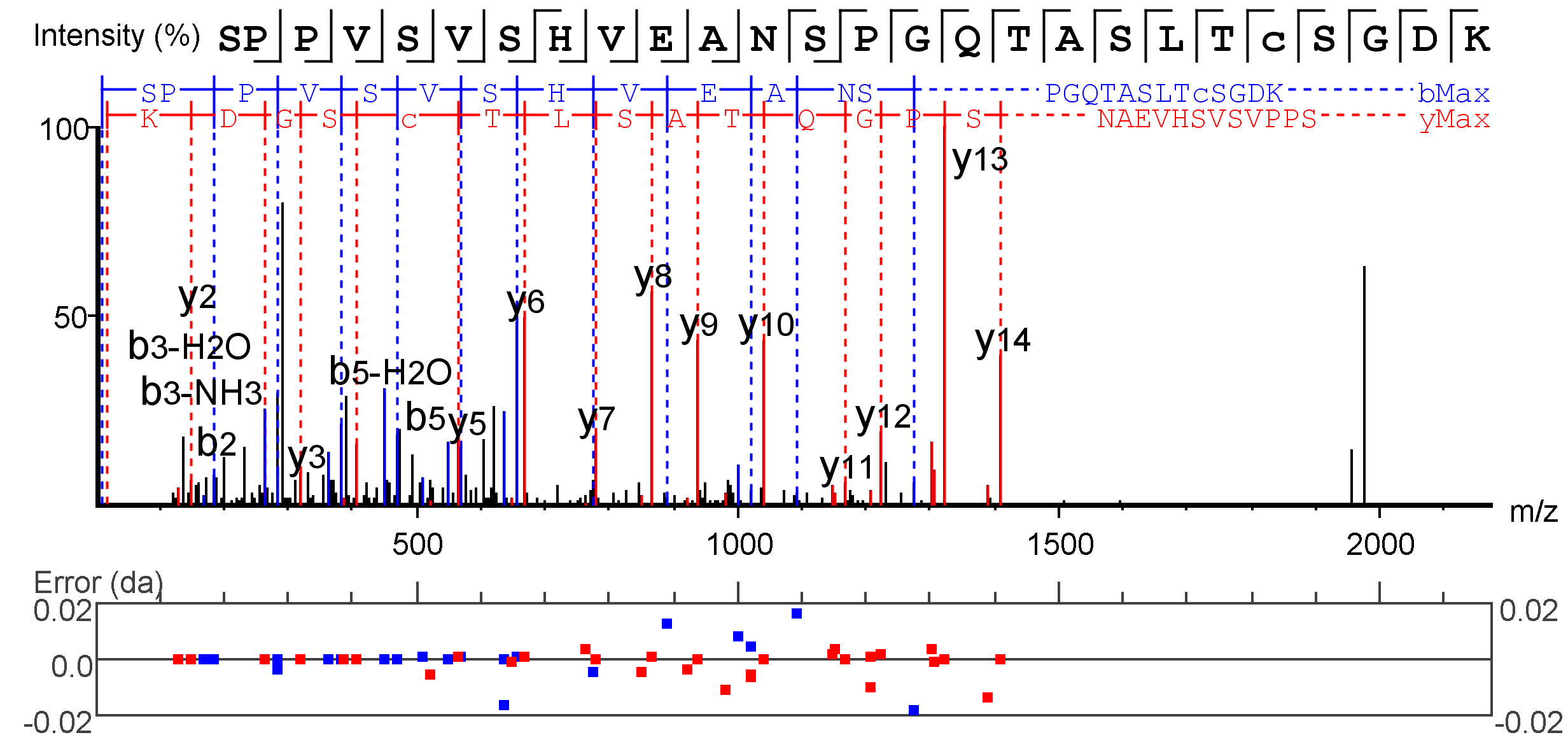


**Supplemental Figure S1**.

1. Patient 5 – Heavy chain


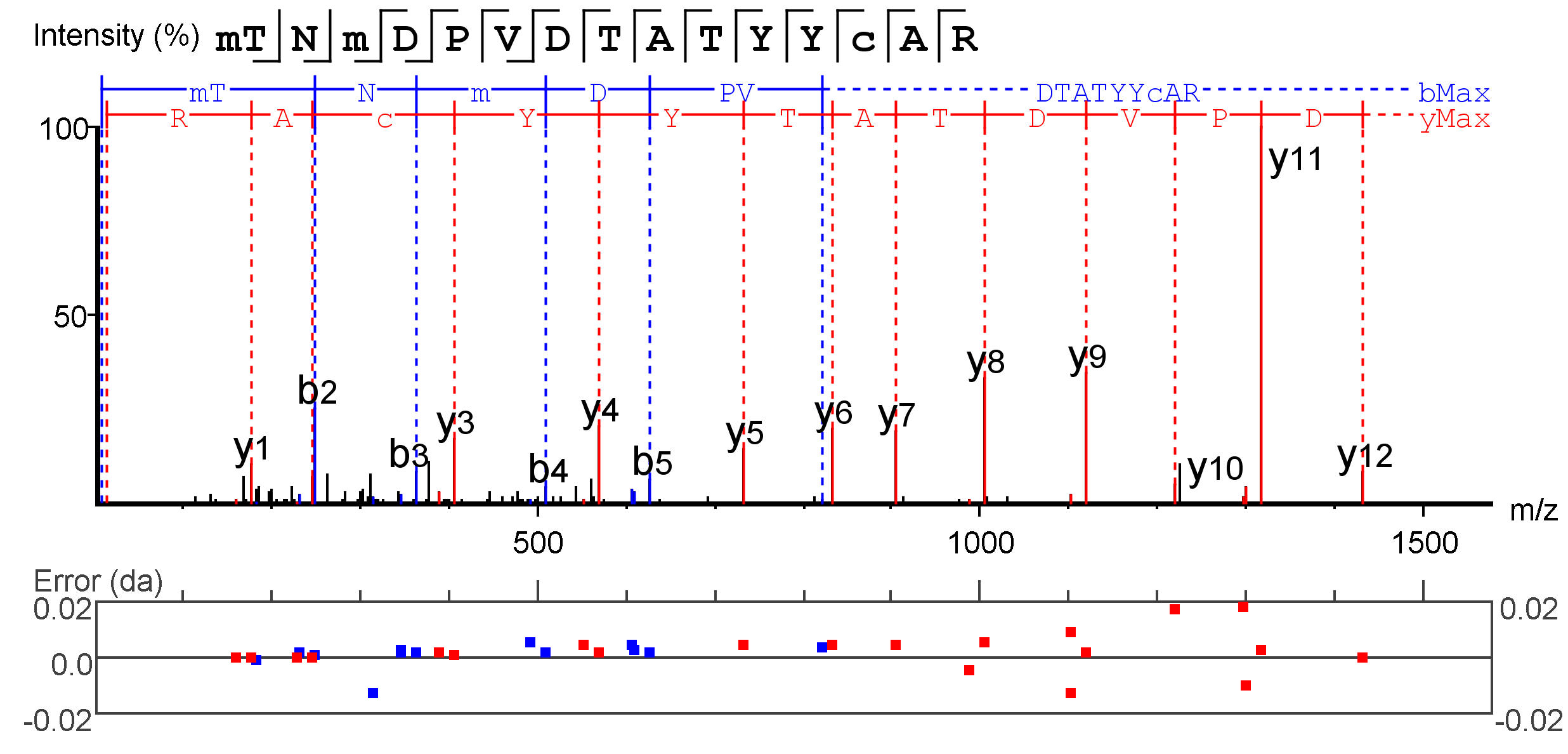


Light Chain


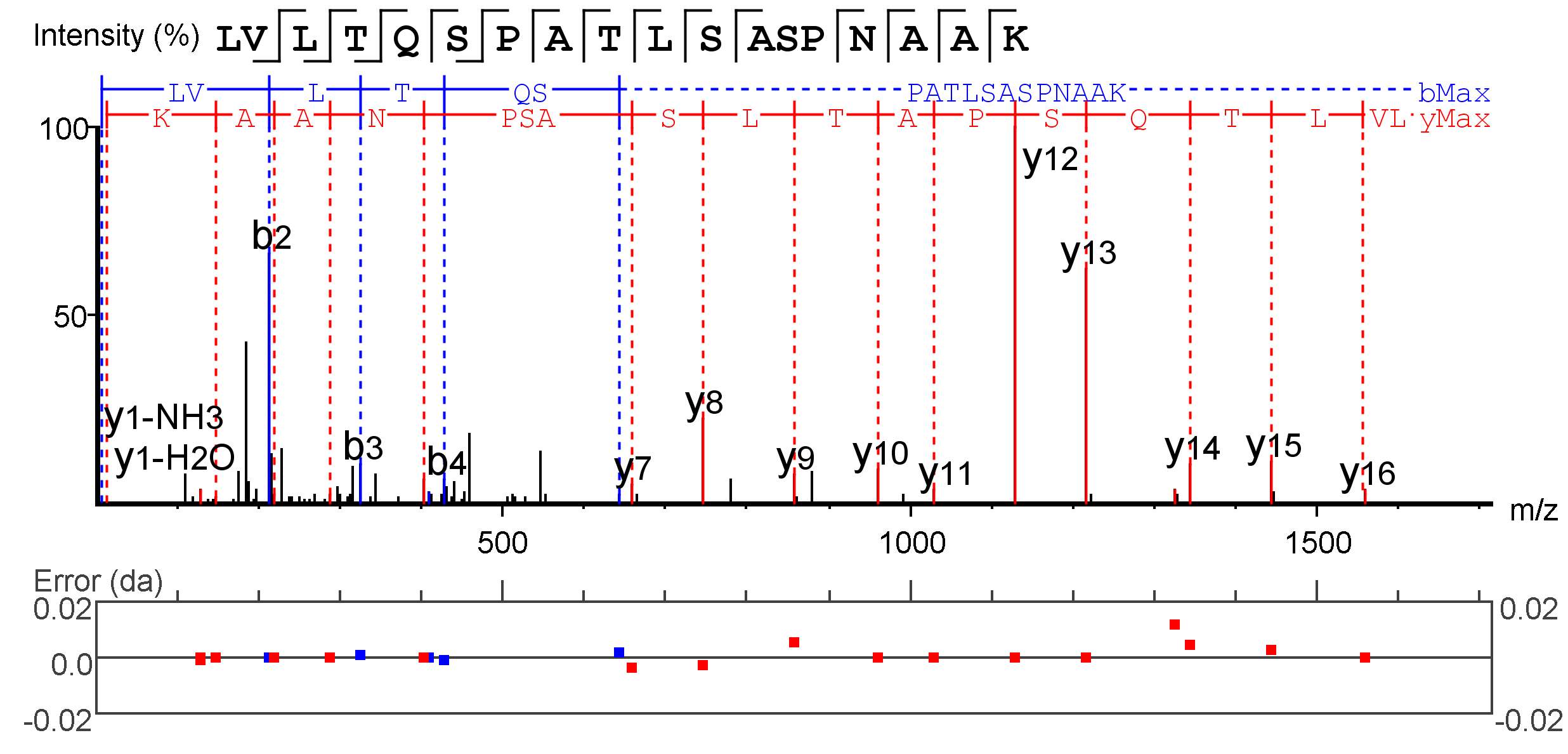


**Supplemental Figure S1**.

1. Patient 6 – Heavy chain


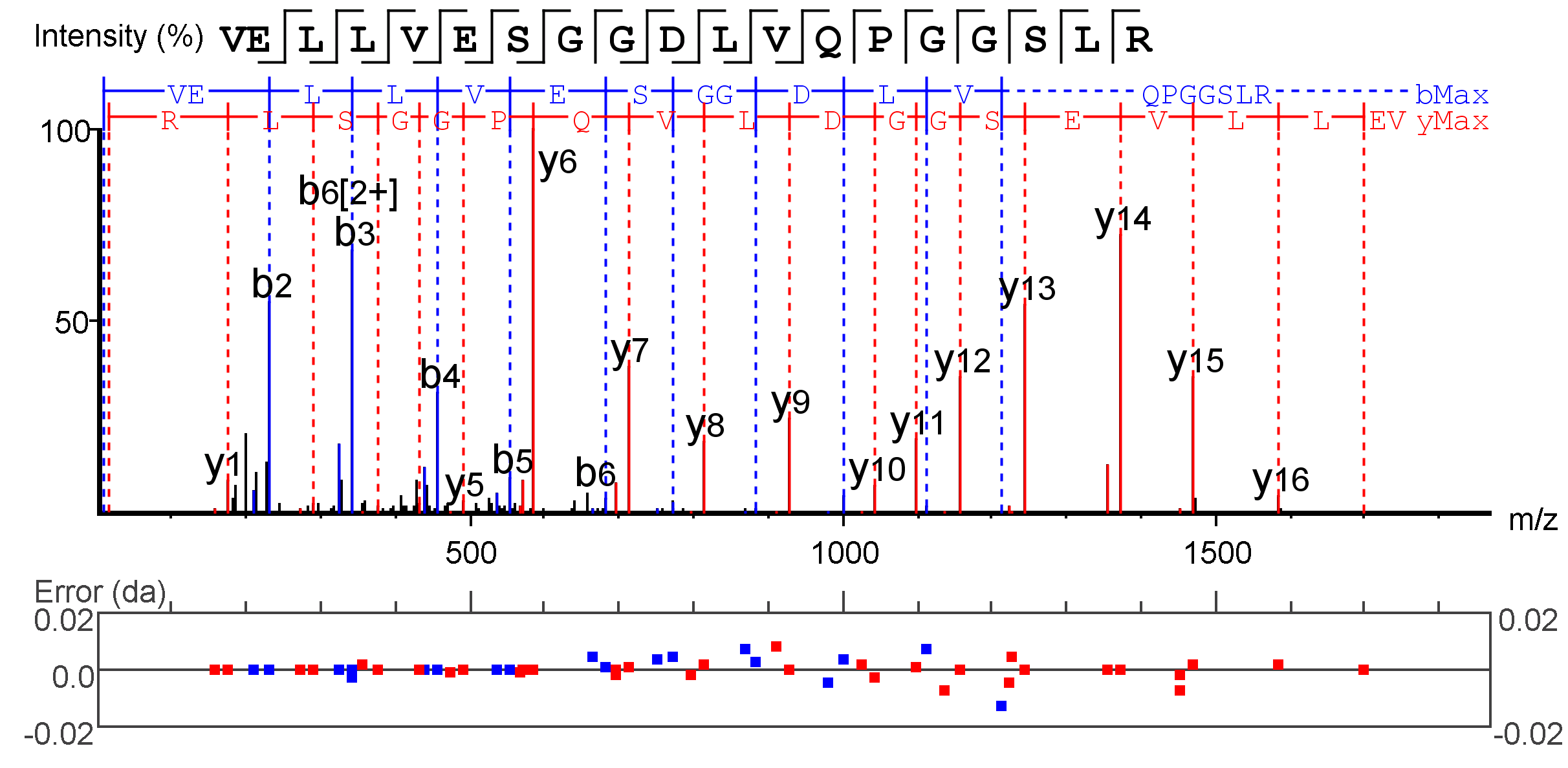


Light chain


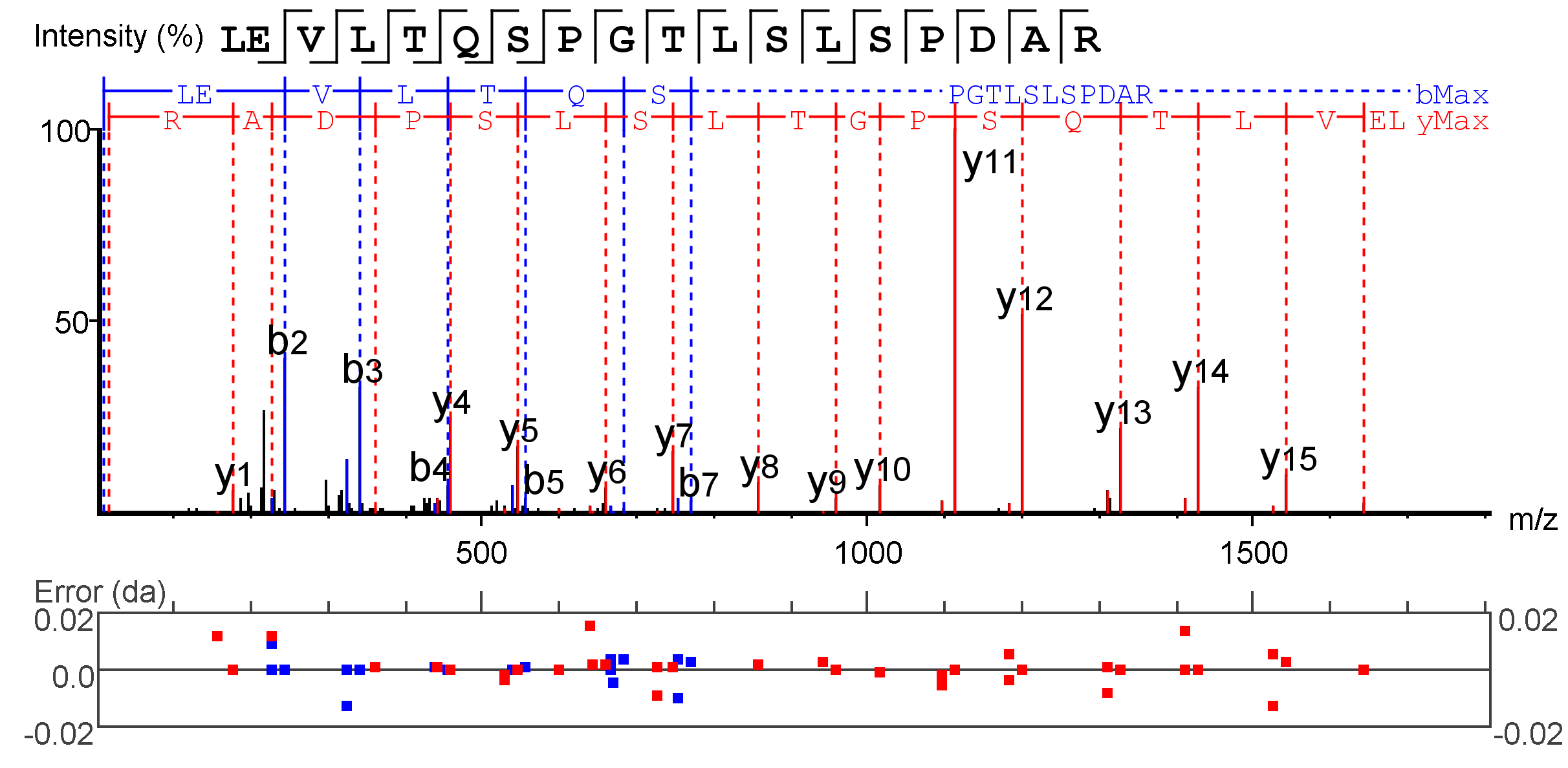


**Supplemental Figure S1**.

1. Patient 7 – Heavy Chain


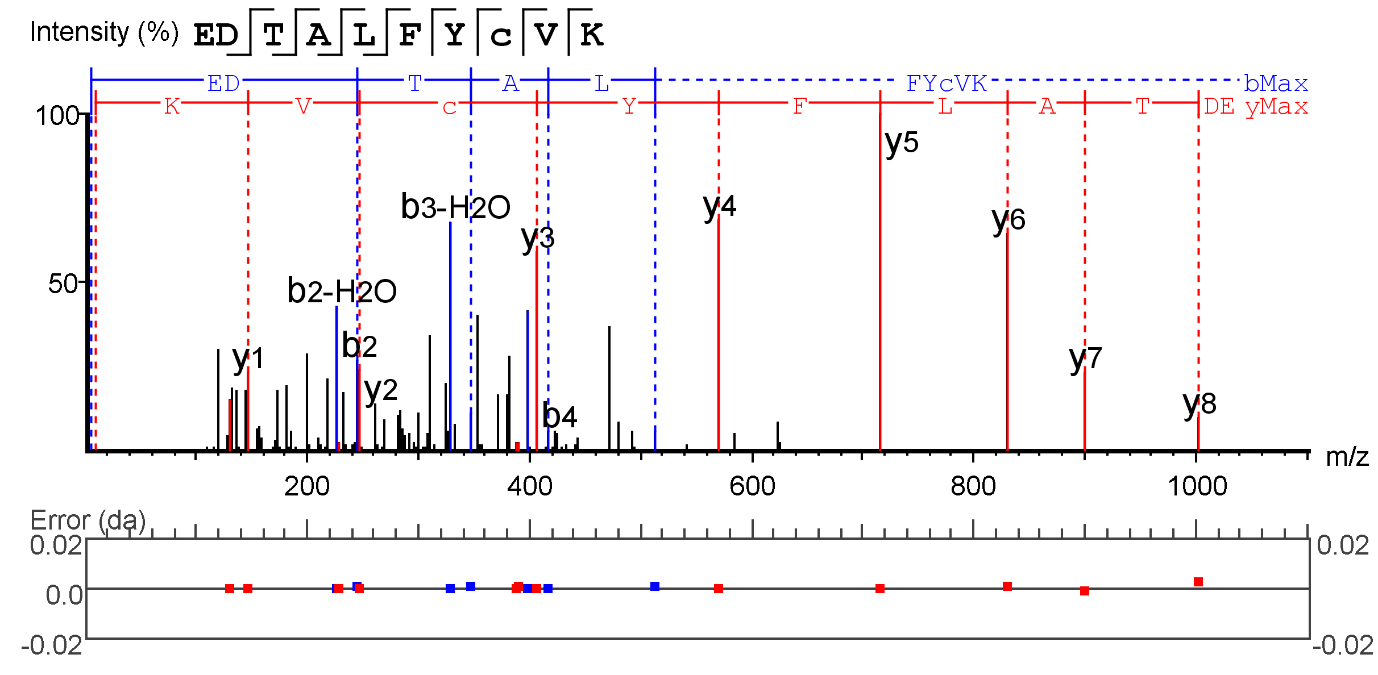


Light chain


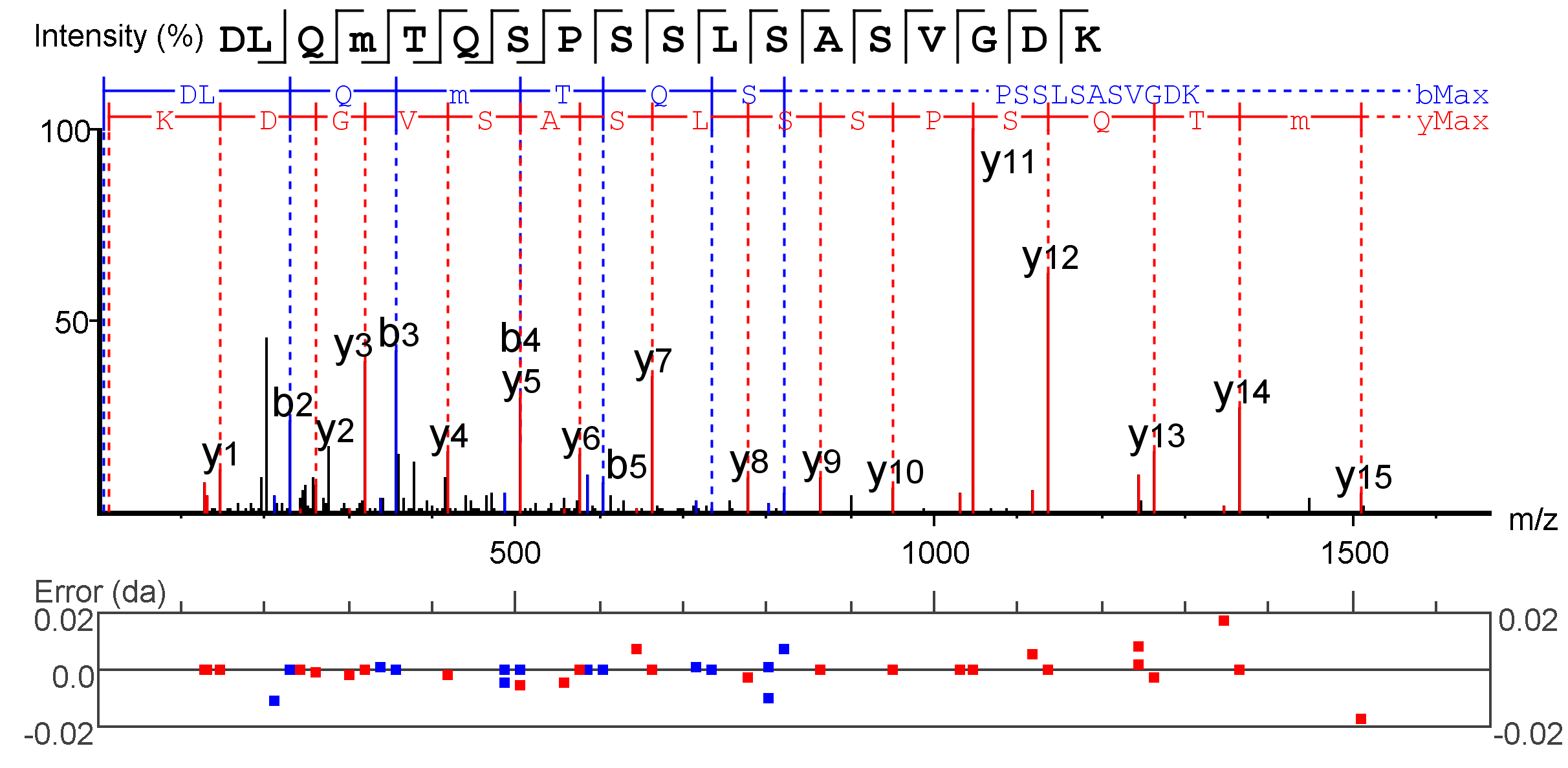


**Supplemental Figure S1**.

1. Patient 8 – Heavy chain


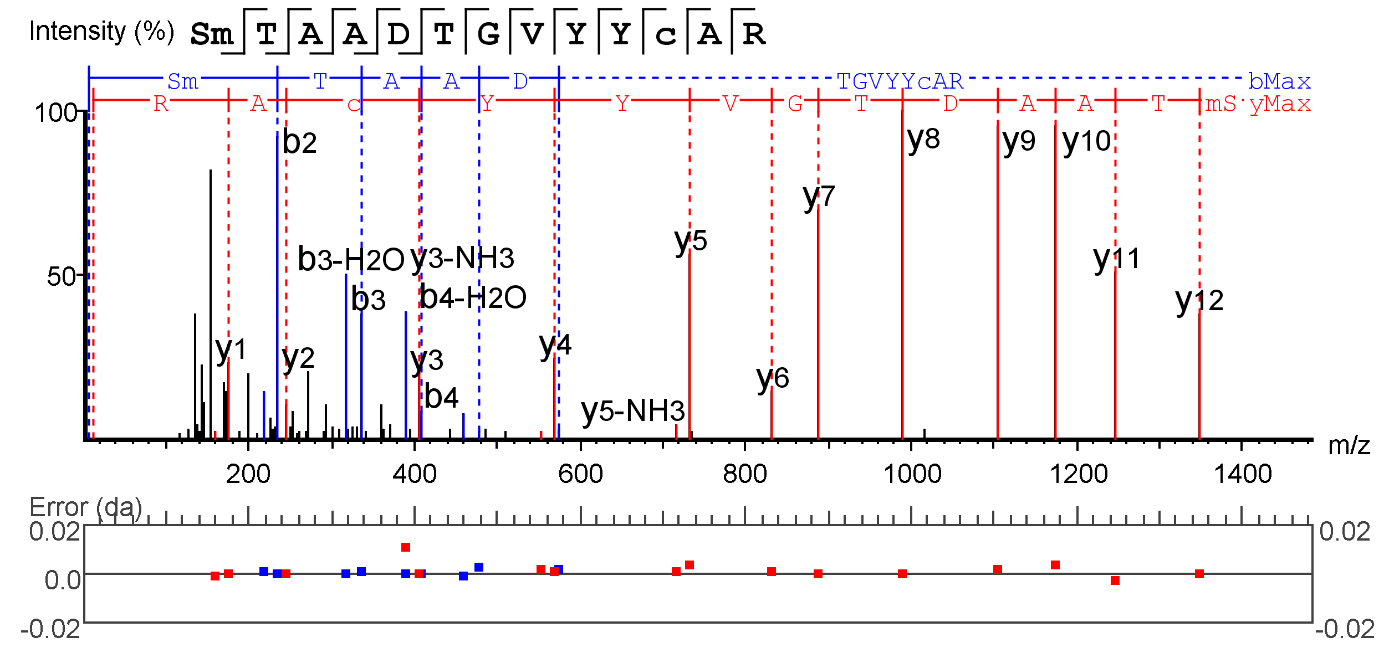


Light chain


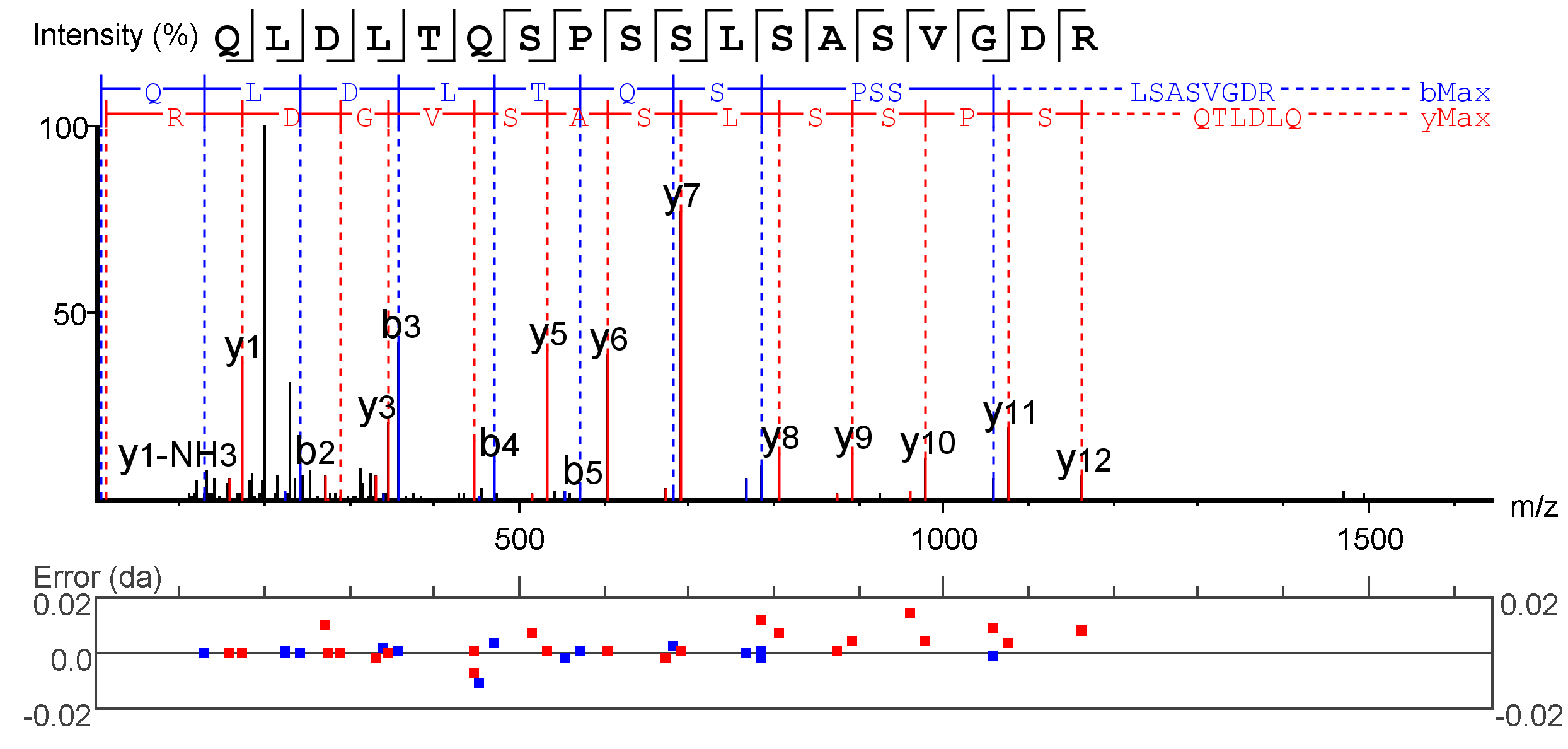


**Supplemental Figure S1**.

1. Patient 9 – Light chain


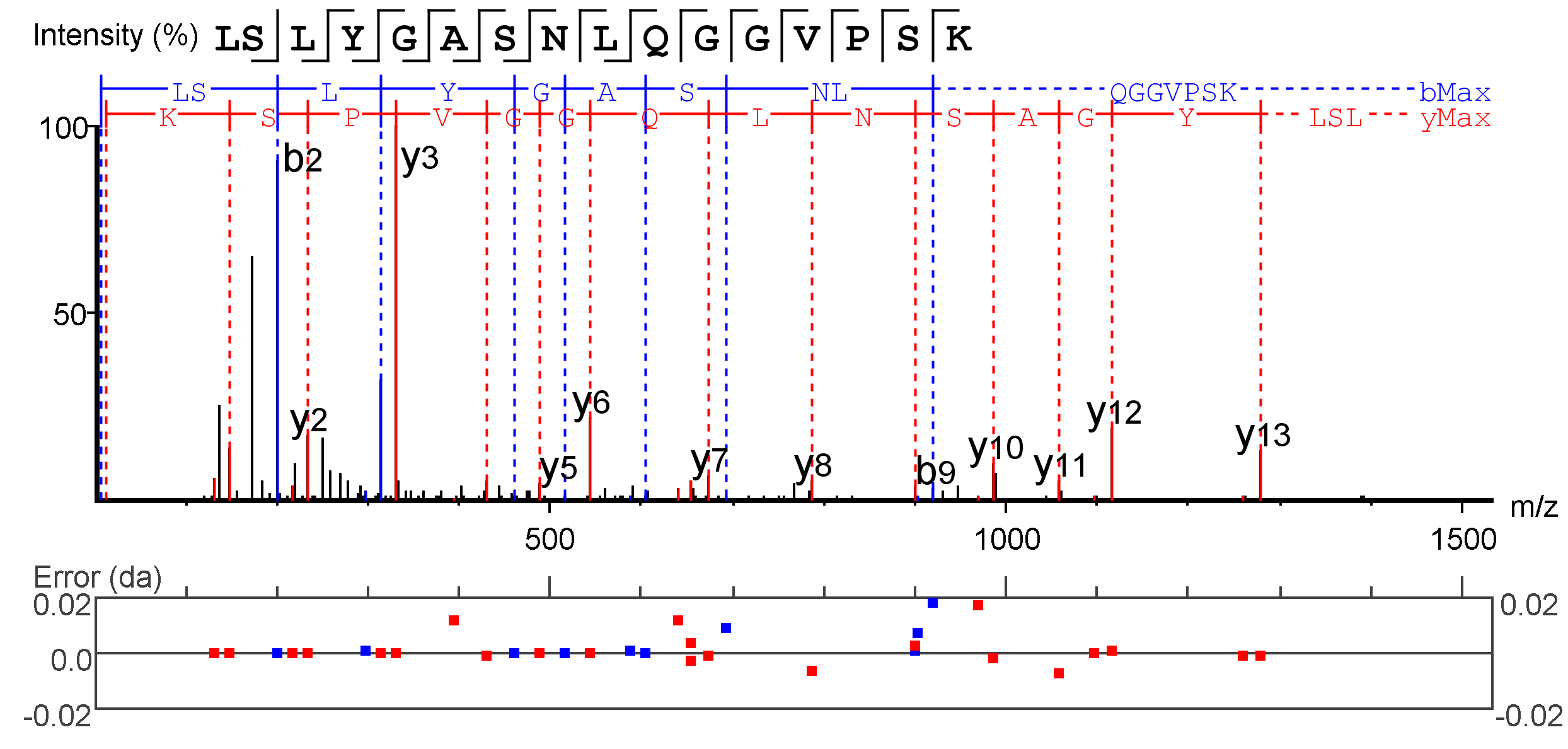


**Supplemental Figure S1**.

1. Reference patient. Heavy chain with DNA reference data available.


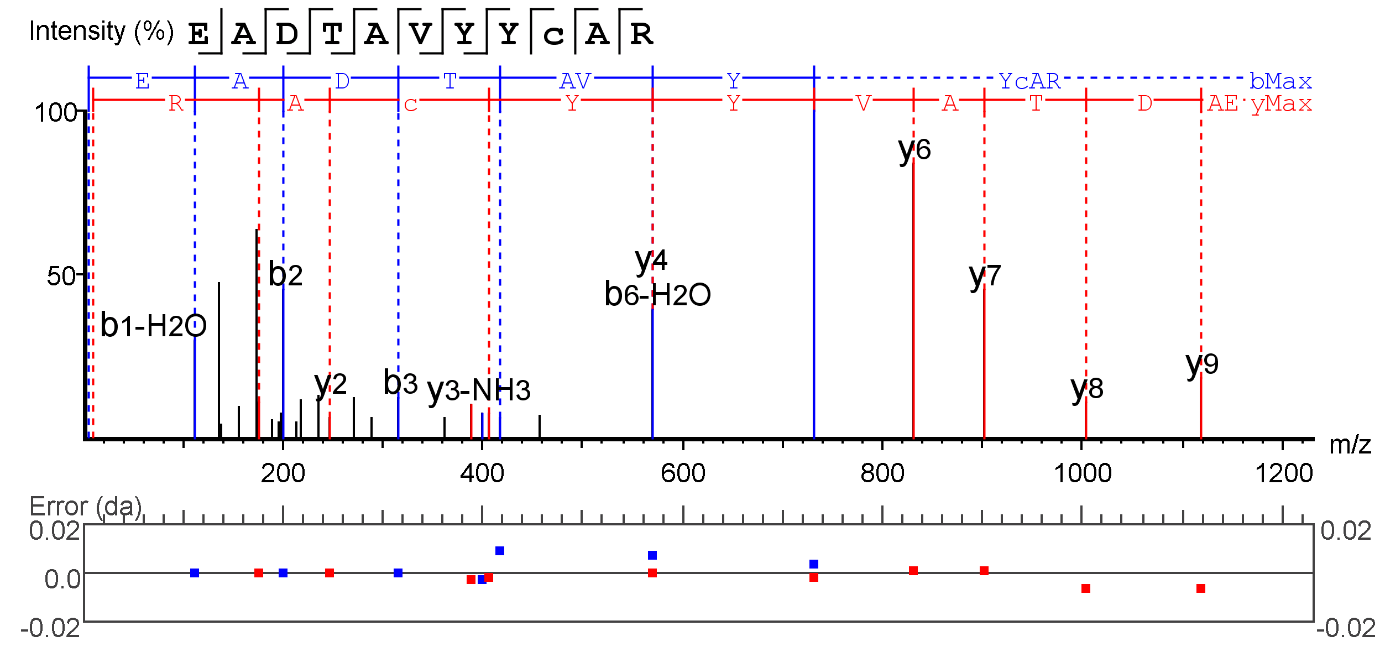


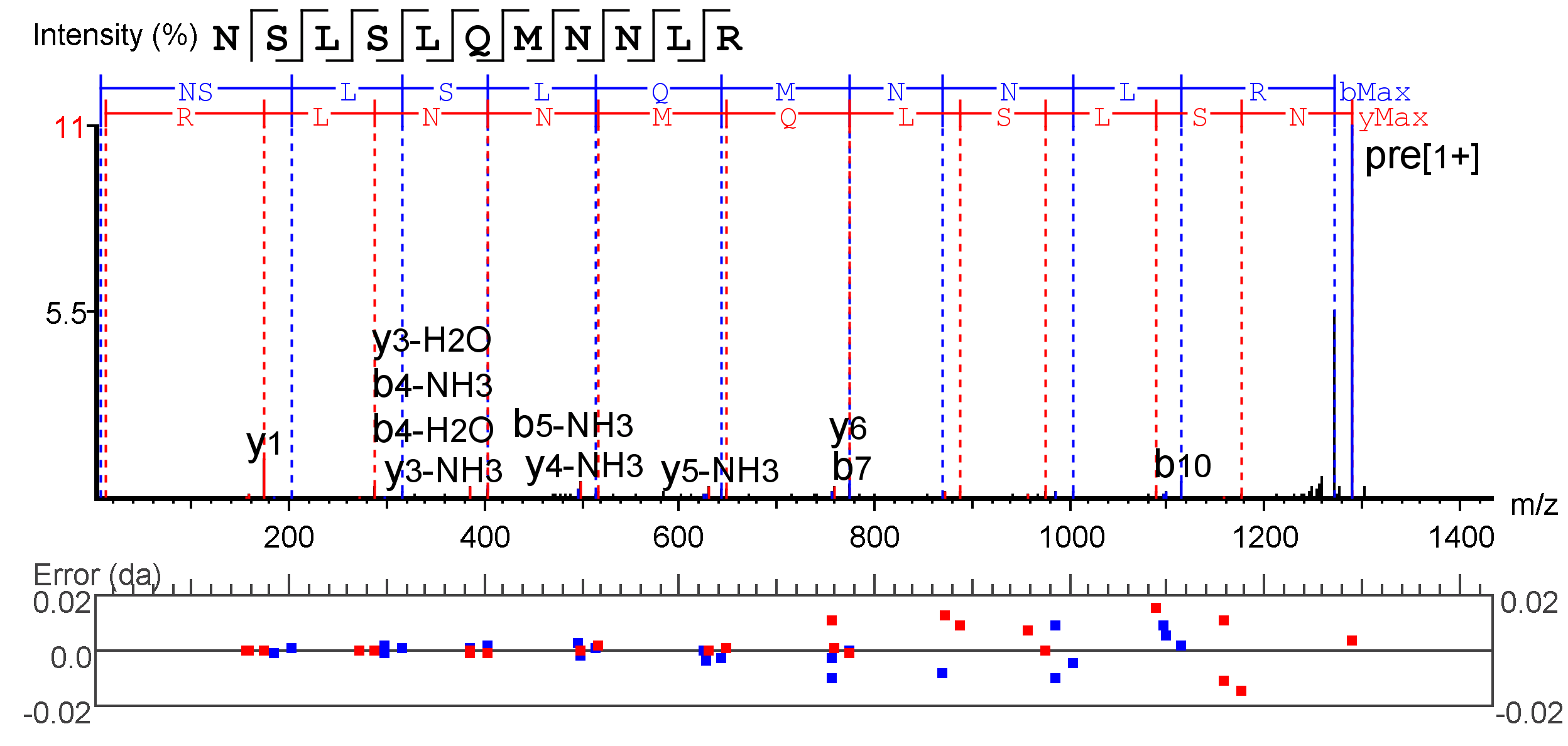

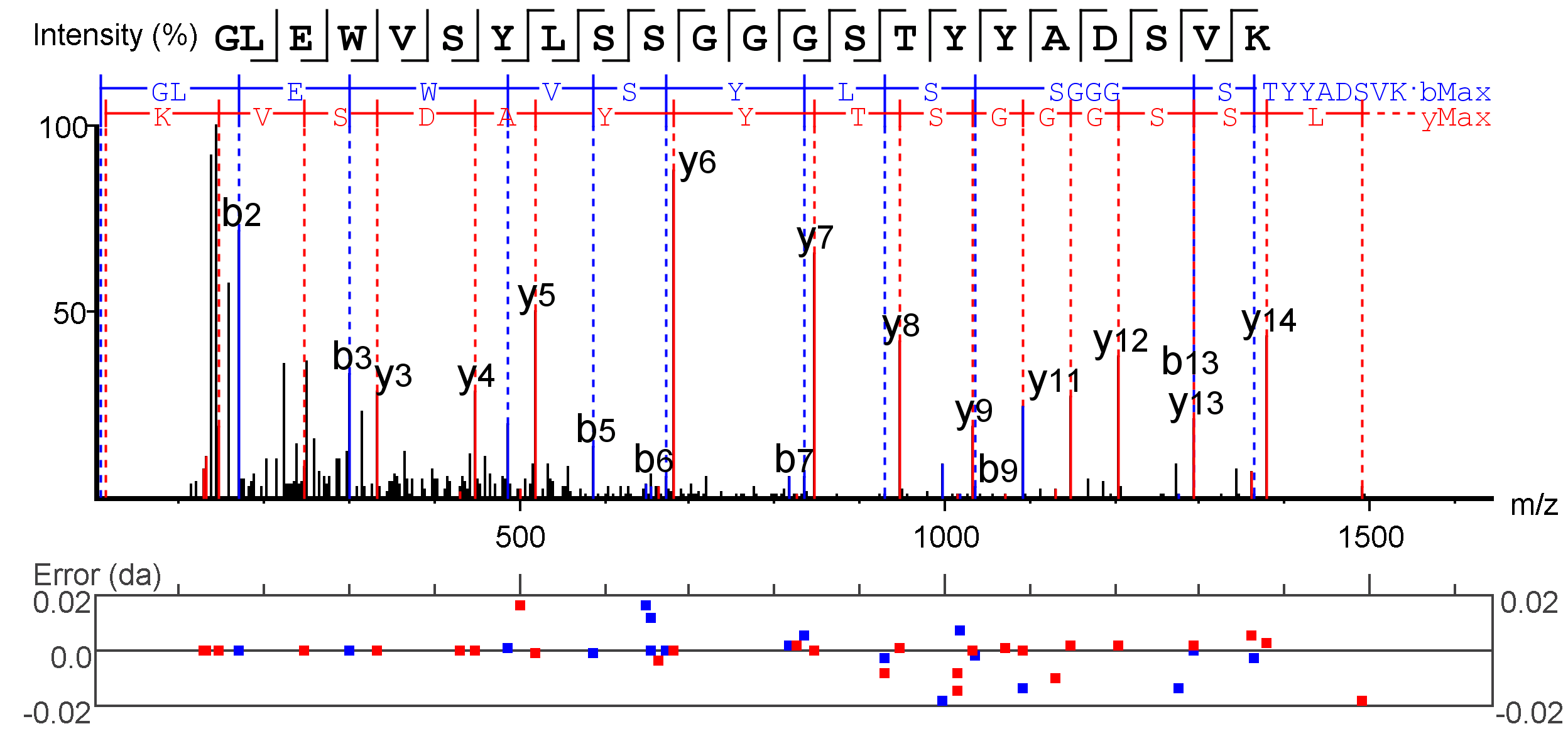
**Supplemental Figure S1**

**Figure S2**.

The dot product between a sample mass spectrum and a reference library spectrum was used as a quality metric in order to assess the fidelity of each result, and to determine whether a sample was positive or negative for M-protein with a cut-off of 0.8. The figure shows examples from Figure 1 annotated with the dot product of all time points (*). The titration in panel D demonstrates the loss of the dot product score when data are below the detection limit. All patient samples in panel A-C exceeded the cut-off for the dot product and were considered positive for the presence of M-protein.


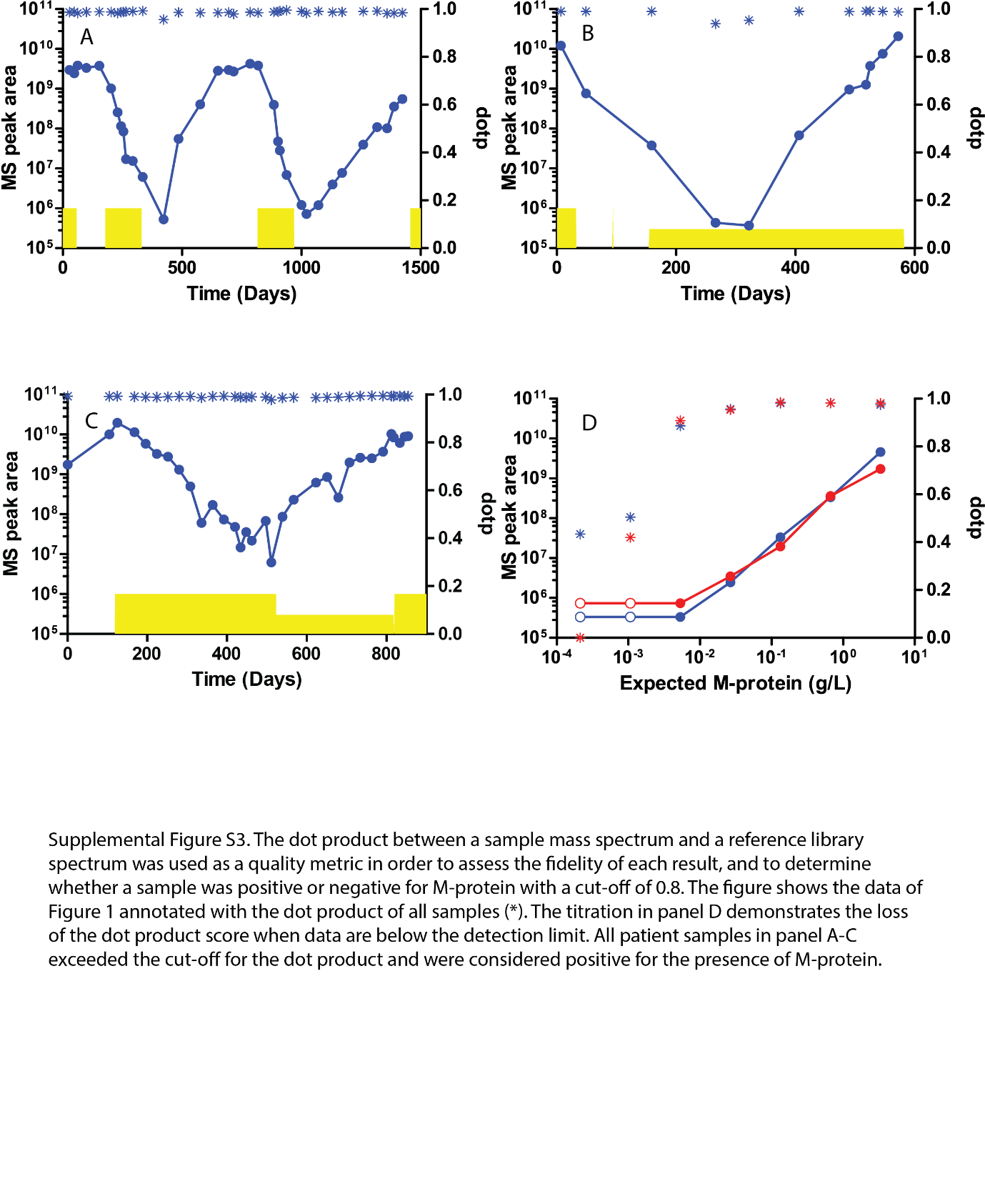


**Figure S3**

M-protein signals for Patient 1 (Figure 2). Dark and light blue traces relate to the same peptide NSVFLEMNSLR, with and without a methionine oxidation respectively. The data in Figure 2 was based on data from the oxidized peptide, but similar disease dynamics were observed with the non-oxidized peptide.


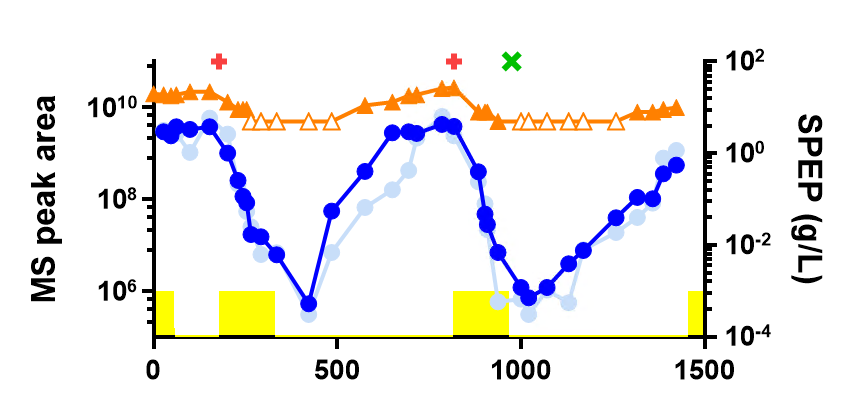

Supplement: Supplementary file 1 [file hs9-6-e758-s001.docx]
